# Acupuncture for Opioid Dependence Patients Receiving Methadone Maintenance Treatment: A Network Meta-Analysis

**DOI:** 10.3389/fpsyt.2021.767613

**Published:** 2021-12-13

**Authors:** Hao Wen, Rouhao Chen, Peiming Zhang, Xiaojing Wei, Yu Dong, Shuqi Ge, Wen Luo, Yiping Zhou, Songhua Xiao, Liming Lu

**Affiliations:** ^1^Department of Neurology, The Sun Yat-sen Memorial Hospital of Sun Yat-sen University, Guangzhou, China; ^2^South China Research Center for Acupuncture and Moxibustion, Medical College of Acu-Moxi and Rehabilitation, Guangzhou University of Chinese Medicine, Guangzhou, China; ^3^Department of Rehabilitation, Zhuhai Hospital of Integrated Traditional Chinese and Western Medicine, Zhuhai, China; ^4^School of Medical Information Engineering, Guangzhou University of Chinese Medicine, Guangzhou, China; ^5^Department of Vasculitis, Affiliated Chinese Medicine Hospital of Guangzhou Medical University, Guangzhou, China

**Keywords:** opioid dependence, acupuncture, methadone maintenance treatment, network meta-analysis, opioid withdrawal

## Abstract

**Objectives:** Opioid dependence has been a threat to public health for hundreds of years. With the increasing number of studies on acupuncture-related therapies for opioid dependence patients receiving methadone maintenance treatment (MMT), its effect of acupuncture therapy in treating MMT patients remains controversial. Therefore, we conducted a multiple-treatments meta-analysis, and incorporated both direct and indirect comparisons, in order to discover the most effective treatment for opioid dependence patients receiving MMT.

**Methods:** Five English databases and three Chinese databases were searched from its inception to August 20, 2020, in order to compare the effects of acupuncture-related therapies and MMT, which was summarized as Western medicine (WM) in the following texts. The quality of studies was assessed according to Cochrane's risk of bias tool 5.1.0, and a pair-wise meta-analysis, cumulative meta-analysis, and the network meta-analysis was performed using the R software (Version 3.6.1) and STATA (Version 14.0). The primary outcome was the effective rate, which was calculated by the ratio of detoxifying patients to the total. The secondary outcome was the Modified Himmelsbach Opiate Withdrawal Scale (MHOWS).

**Results:** A total of 20 trials were included, which consisted of comparisons among WM, traditional Chinese medicine (TCM), and the four types of acupuncture, namely, manual acupuncture (MA), electro-acupuncture (EA), auricular acupuncture (AA), and transcutaneous electrical acupoint stimulation (TEAS). Though none of the trials were at low risk of bias. In the pair-wise meta-analysis, no statistically significant differences were observed in terms of the effective rate. Furthermore, MA was more efficacious than WM, EA, and TEAS in MHOWS, with mean differences (MDs) of (−8.59, 95% CI: −15.96 to −1.23, *P* < 0.01), (−6.15, 95% CI: −9.45 to −2.85, *P* < 0.05), and (−10.44, 95% CI: −16.11 to −4.77, *P* < 0.05), respectively. In the network meta-analysis, MA was more effective than WM (RR: 1.40, 95% CI: 1.05 to 1.99) on the effective rate, and (MD: −5.74, 95% CI: −11.60 to −0.10) on MHOWS. TEAS was more effective than WM (MD: −15.34, 95% CI: −27.34 to −3.46) on MHOWS. Synthetically, MA had the highest probability to rank first in treating opioid dependence.

**Conclusions:** The existing evidence shows that acupuncture related-therapies may effectively be used for treating patients receiving MMT, and that manual acupuncture may be the best choice for opioid dependence among all kinds of acupuncture-related therapies. Nevertheless, reducing the relapse and promoting the recovery of opioid dependence need more efforts from not only the medical industry but also government support, security system, and educational popularization. To strengthen the assurance of acupuncture-related therapies in the treatment of opioid dependence, we expected that clinical trials with high quality would be conducted, to provide more confident evidence.

## Introduction

According to the World Drug Report 2018, ~31 million people are affected by opioid-use disorders, which cause the greatest burden of severe disease and drug-related deaths worldwide. In China, ~2.5 million people use illicit drugs, and opioid and methamphetamine represent the majority. In order to solve this prominent public health problem, methadone has been widely used to suppress withdrawal symptoms induced by the abrupt discontinuation of drugs, decrease the use of opioids and other illicit drugs, and reduce criminal activities. At present, opioid agonist treatment has effectively reduced the incidence of HIV and hepatitis C caused by sharing needles (1). Patients with opioid dependence must receive opioid substitution (e.g., methadone maintenance treatment (MMT), buprenorphine, and clonidine) in order to control the cravings for heroin for years, or even throughout their entire life.

Although MMT has achieved some beneficial effects, its side effects have been widely reported (e.g., constipation, dizziness, drowsiness, and weakness), especially during the first several weeks of methadone stabilization. Patients often complain of insomnia and cravings during MMT, which may affect patient compliance with MMT and contribute to the risk of relapse. Furthermore, as an artificial opioid compound, the long-term use of methadone can impair patients' cognitive function and sustained attention and reduce the striatal dopamine transporter function. Although the use of MMT prolongs the dependence period, this may create new drug dependence at the same time (2). Hence, a broad variety of non-pharmacological therapies are used against opioid dependence. As a non-pharmacological therapy, acupuncture has been recommended to treat substance dependence by the World Health Organization. However, it remains controversial whether acupuncture therapy should be used in the clinical treatment of opioid dependence patients receiving MMT. Thus, comprehensive analysis and evaluation of the relevant evidence are still required. We systematically collected the evidence of acupuncture for the treatment of opioid dependence patients and conducted a pooled analysis, in order to assess the efficacy of acupuncture therapy in the treatment for opioid dependence patients receiving MMT.

## Materials and Methods

A systematic review was conducted following the general principles outlined in the Center for Reviews and Dissemination (CRD) Guidance and the PRISMA statement. We registered this systematic review in the PROSPERO database (CRD: 42021233950).

### Search Strategy

We searched 8 professional digital databases from their inception to August 2020: Medline, EMBASE, Cochrane Library, SCOPUS, Web of Science, CNKI, VIP, and Wanfang. A search term and search strategy were developed for each database (search term in Supplementary Files), and all articles were entered into the NoteExpress Bibliography Software. Two researchers reviewed the search hits by reading the titles and abstracts, deleting the duplicates or non-clinical research, and sorting out the classifications according to the criterion.

### Study Selection and Intervention Definitions

#### Study Design

The present network meta-analysis included randomized controlled trials (RCTs) published in the Chinese or English language, and the effects of different kinds of acupuncture therapies in opioid dependence were evaluated.

#### Participants

We intended to include adult participants. However, it was estimated that opioid dependent patients were emerging in a younger population. Hence, we adjusted the lower bound of age to 16 years old. These enrolled participants were diagnosed with opioid dependence according to the Chinese Classification and Diagnostic Criteria of Mental Disorders (CCMD) or Diagnostic and Statistical Manual of Mental Disorders (DSM). Based on the diagnosis criterion, participants were recruited when they had the following: (1) opioid abuse history; (2) drug dependent syndrome, withdrawal symptoms, or personality change after taking opioids; (3) impaired social function or even criminal behaviors due to drug-taking; and (4) mental health disturbance caused by opioid (3). Pregnant women, infectious people, and participants over 60-years-old were excluded.

#### Interventions

In the regular detoxification therapy, opioid dependent people were given methadone maintenance treatment as substitution or harm reduction therapy, which aimed to make progress for addicted people from maintenance to detoxification and then abstinence (4). Considering the basic effect of MMT in opioid dependence, patients in both the experimental groups and control groups were all taking MMT as usual care. Manual acupuncture, electro-acupuncture, auricular acupuncture, and other acupuncture-related therapies were eligible as experimental intervention for inclusion, while the control groups were taking MMT only.

#### Outcomes

We considered RCTs that reported the following outcome measures: (1) recovery rate or effective rate, assessed by the quantity of participants, who were completely detoxified, nearly detoxified, and partially detoxified from the therapy; (2) withdrawal symptoms scores measured by the Modified Himmelsbach Opiate Withdrawal Scale (MHOWS) (5). In effective rate, we defined a patient was “completely detoxified” when the withdrawal syndrome was exhaustively disappeared (100%), with normal laboratory test results, and no craving for opioid. If a patient was “nearly detoxified,” which represented a 70% alleviation of withdrawal syndrome, with significant improvement of laboratory tests. “Partially detoxified” indicated a 30% relief of withdrawal syndrome and an improvement of laboratory tests. In the MHOWS, we defined “effectiveness” when a patient scored <12 and had a negative urine morphine test. MHOWS consisted of 10 non-accurately measurable and 4 accurately measurable signs of withdrawals. For these 10 non-accurately measurable categories, 1 point was added for any of yawning, lacrimation, rhinorrhea, or perspiration, 3 points were added for any of tremor, gooseflesh, anorexia, or dilated pupils, 5 points were added for restlessness in any 1 day, and 5 points were added for each emesis. Each subject was assigned a weighted point value, and the value depended on the presence or severity of the symptom. The four measurable subjects followed the MHOWS morphine agonist baseline evaluations for temperature, respiratory rate, weight, and systolic blood pressure: 1 point was added for each 0.1°C rise in temperature, 1 point was added for each respiration per minute increase, 1 point was added for each 2-mmHg increase (up to 30 mm) in systolic B/P, and 1 point was added for each pound loss in weight. A lower score on the MHOWS corresponded to fewer objective withdrawal symptoms.

### Assessment of Risk of Bias

In order to evaluate the methodological quality of included studies, two reviewers (S.G. and W.L.) independently assessed the risk of bias according to Cochrane's risk of bias tool 5.1.0 (6). There was a total of seven domains: (1) Was the allocation sequence random? (2) Was the allocation sequence concealed until participants were enrolled and assigned to interventions? (3) Were participants aware of their assigned intervention during the trial? (4) Were carers and people delivering the interventions aware of participants' assigned intervention during the trial? (5) Was there a report on the completion of the data? (6) Was there selective reporting in the results? (7) Was there potential bias in the study? Each domain was assessed as one of the following levels: “low risk of bias,” “unclear risk of bias,” and “high risk of bias.” These corresponded to the following: “definitely yes,” “probably yes,” and “definitely no,” respectively. Any disagreements were resolved by discussion with a third reviewer (H.W.). We judged a study based on the methodological information of randomization, allocation concealment, blinding, outcome completion, selective reporting, and other potential factors. If four or more items were assessed as “low risk of bias,” the study was evaluated as high quality.

### Data Extraction

Two reviewers (X.W. and Y.D.) screened the full texts, extracted the correlated information from all the included studies, and cross-checked the information, in order to ensure the consistency and accuracy of the extraction. We extracted the general information (e.g., publication year, author, sample size, gender, age, and outcome measures), drug-abuse circumstances (e.g., duration, designations, drug-taking ways, and daily intake), and information of interventions (e.g., the designations and dosage of opioid substitution, and the types, usage, frequency, intensity, and duration of acupuncture-related therapies). The information above was concluded in a study characteristics table.

### Statistical Analysis

A head-to-head meta-analysis was performed to evaluate the efficacy of acupuncture-related therapies and opioid substitutions in opioid dependence. The quality of studies was assessed according to Cochrane's risk of bias tool 5.1.0. We used R software (Version 3.6.1) to conduct the pair-wise meta-analyses and cumulative meta-analyses. The meta packages were used for pair-wise meta-analyses with random-effects model command, “metabin” command for dichotomous data while “metacont” command for continuous data. The effective rate was reported as risk ratio (RR), and for continuous data, the MHOWS was reported in mean difference (MD). All reported data corresponded with the 95% confidence intervals (CIs). The cumulative meta-analyses were carried out using “metacum” command, verifying the results of meta-analyses.

Two computer software, R software (Version 3.6.1) (7) and STATA (Version 14.0) (8), were used to complete the correlated analysis and plot the graph. For R, we initially calculated this using the gemtc (Version 0.8-2) package (9). A Bayesian inference with Markov chain Monte Carlo (MCMC) simulation was performed in RJAGS (Version 4.3.0) (10), inferring the posterior probability based on the prior probability. The estimation and deduction were carried out when the convergence was stable. Then, a network meta-analysis was conducted in STATA (11).

We constructed a fixed-effect model and a random-effect model, and set the parameter values as follows: n.chain = 3, factor = 2.5, n.adapt = 5,000, and n.iter = 20,000. Three chains yielded 20,000 iterations and the factor of 2.5.

Based on the deviance information criterion (DIC) from R and the diagnostic parameters potential scale reduction factor (PSRF) from Brooks-Gelman-Rubin, we judged the comparison between the fixed-effect model and random-effect model. If the PSRF value was close to 1 and the DIC value was lesser, the convergence of the model was more suitable.

The node-splitting analysis (12) helped to generate the inconsistency test. If the *P*-value of the node-splitting analysis was >0.05, the direct evidence was consistent with the indirect evidence. Inconsistency was defined as the differentiation between the direct and indirect evidence, with a *P* < 0.05. These analyses were performed after the derivation of the inconsistency was determined (13). The rank of interventions was assessed by calculating the surface under the cumulative ranking curve (SUCRA) (14), and shown in the established Rankograms. The ranking probability was correlated to the surface under the curve. A larger surface represented a higher probability for intervention to rank first among the therapies.

## Results

### Study Selection and Characteristics

**Figure 1:**
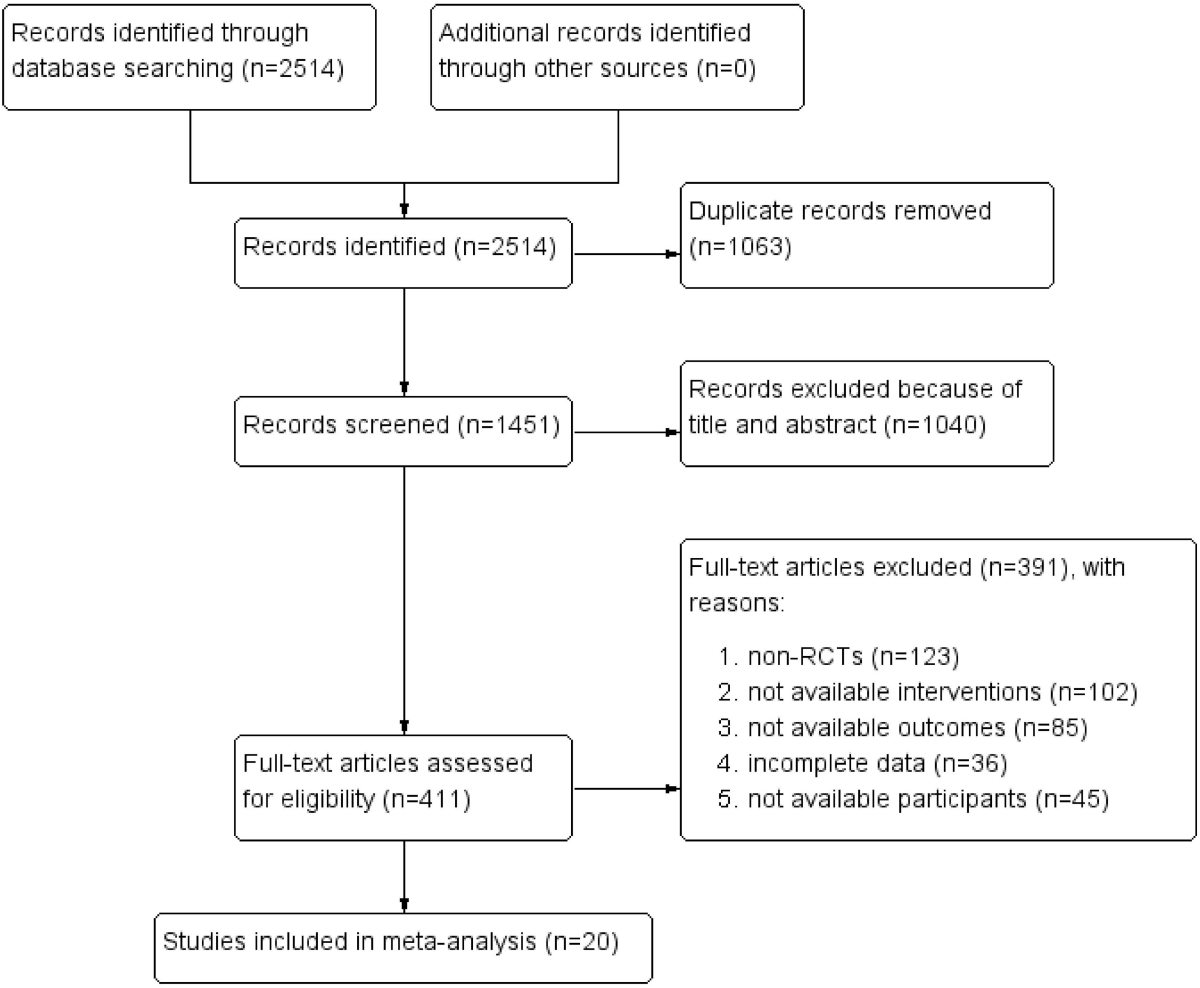
PRISMA flow diagram.

A total of 2,154 results were collected from eight digital databases using the search strategy. A total of 411 applicable full-text results were evaluated for eligibility, and 391 records were excluded, based on the following: (1) non-randomized studies (*n* = 123); (2) incomplete data (*n* = 36), ineligible outcomes (*n* = 85), interventions (*n* = 102), and participants (*n* = 45). The flowchart for the trial selection is shown in Figure 1, and the main characteristics of the included trials are summarized in Table 1. All selected trials were conducted in China and published from 1997 to 2010 in the Chinese language. The involved patients were diagnosed with opioid dependence derived from CCMD-2-R, CCMD-III (35), DSM-III-R, and DSM-IV (36).

**Table 1:** Characteristics of included studies.

**Study**	**Arms**	**Group**	**Sample size (*n*)**	**Sex (male/female)**	**Age (y)**	**Course**	**Interventions**	**Acupoint**	**Duration (d)**	**Follow-up**	**Outcome measures**	**Diagnostic criteria**
Wang et al. (15)	2	G1	100	86/14	18–39	6–60 m	MA+MMT	LR3, KI3, SP6	10	6 m	➀	CCMD-2-R
		G2	100	87/13	17–38	5–60 m	MMT	/				
Zeng et al. (16)	2	G1	31	26/5	33.2 ± 5.5	6.00 ± 2.82 y	MA+MMT	DU20, DU14, DU11, DU10, DU9, DU4	10	NA	➁	DSM-III-R
		G2	26	21/5	34.2 ± 4.8	6.23 ± 2.93 y	MMT	/				
Zhang et al. (17)	3	G1	28	17/11	27.5 ± 4.8	32.4 ± 15.16 m	MA+MMT	PC6, ST36, SP6	7	NA	➀➁	DSM-IV
		G2	30	19/11	27.6 ± 4.6	40.56 ± 16.08 m	MMT	/				
		G3	23	15/8	28.1 ± 7.1	29.52 ± 18.12 m	TCM+MMT	/				
Wang et al. (18)	2	G1	60	49/11	17–39	0.5–4 y	AA+MMT	CO10, CO13, CO14, CO18, AT4, TF4, AH6a	10	6 m	➀	CCMD-2-R
		G2	60	51/9	16–38	0.5–4 y	MMT	/				
Jin (19)	2	G1	32	NA	18–42	63 m	AA+MMT	TF4, CO10, CO12, CO13, CO14, CO15	28	3 m	➀	CCMD-2-R
		G2	30	NA	18–42	63 m	MMT	/				
Liu et al. (20)	2	G1	74	68/6	16–46	6.20 y	EA+MMT	DU19, DU20, EX-HN5, LI11, PC6, ST36, GB34, HT7	10	NA	➁	CCMD-III
		G2	74	66/8	17–45	5.90 y	MMT	/				
Zhang et al. (21)	2	G1	43	39/4	16–51	8.20 y	EA+MMT	DU19, DU20, EX-HN5, LI11, PC6, ST36, GB34, HT7	10	NA	➁	CCMD-2-R
		G2	43	40/3	18–49	7.96 y	MMT	/				
Yuan et al. (22)	2	G1	60	51/9	18–40	0.5–6 y	EA+MMT	LR3, KI3, SP6	10	NA	➀	CCMD-2-R
		G2	60	52/8	17–39	0.5–5.5 y	MMT	/				
Rong and Liu (23)	3	G1	33	NA	18–60	7.36 ± 4.54 y	MA+MMT	MS1, MS5, MS7, MS2, MS8, MS11	10	NA	➁	DSM-IV
		G2	31	NA	18–60	7.10 ± 3.28 y	EA+MMT	EX-HN1, PC6, LI4, ST36, SP6				
		G3	30	NA	18–60	7.10 ± 4.09 y	MMT	/				
Niu et al. (24)	2	G1	66	NA	18–40	6 m-3 y	AA+MMT	CO10, CO12, CO14, CO15, CO18, TF4, AT4, AH6a	90	NA	➀	CCMD-2-R
		G2	66	NA	18–40	6 m-3 y	MMT	/				
Wan et al. (25)	2	G1	89	65/24	25.4	1.5 y	AA+MMT	CO10, CO15, CO18, AT4, AH6a, TF4	10	20 d	➀	DSM-IV
		G2	60	51/9	24.3	1.4 y	MMT	/				
He and Li (26)	2	G1	70	NA	23 ± 5	NA	EA+MMT	NA	15	6 m	➀	CCMD-III
		G2	70	NA	23 ± 5	NA	MMT	/				
Song et al. (27)	2	G1	30	18/12	27.4 ± 6	32.6 ± 18.1 m	MA+MMT	PC6, LI4, SP6	7	NA	➀➁	DSM-IV
		G2	30	19/11	27.6 ± 4.6	40.6 ± 16.1 m	MMT	/				
Chen (28)	2	G1	34	NA	18–45	1.5–22 y	EA+MMT	PC6, PC8, LI4, ST36, SP6	30	6 m	➀➁	DSM-IV
		G2	34	NA	18–45	1.5–22 y	MMT	/				
Zong et al. (29)	3	G1	20	16–48	10/10	35.7 m	EA+MMT	RN12, EX-HN3, SP6, PC6, EX-B2	20	NA	➁	DSM-IV
		G2	23	16–48	9/14	31.5 m	TCM+MMT	/				
		G3	28	16–48	15/13	26.9 m	MMT	/				
Wu et al. (30)	3	G1	30	NA	18–60	NA	MA+MMT	EX-HN1, PC6, LI4, ST36, SP6	10	NA	➁	CCMD-2-R
		G2	30	NA	18–60	NA	MMT	/				
		G3	30	NA	18–60	NA	TEAS+MMT	LI4, PC8, ST36, SP6				
Zhao et al. (31)	2	G1	56	NA	28.6 ± 7.0	12.46 ± 18.64 m	EA+MMT	SJ5, PC6, PC8, LI4	10	NA	➀	DSM-IV
		G2	56	NA	28.6 ± 7.0	12.46 ± 18.64 m	MMT	/				
Wang (32)	2	G1	40	30/10	17–51	8.11 y	TEAS+MMT	PC6, LI11, ST36, GB34, HT7	10	NA	➀	CCMD-2-R
		G2	25	21/4	18–49	7.81 y	TCM+MMT	/				
Liu et al. (33)	2	G1	20	NA	26.9+5.5	NA	EA+MMT	NA	14	NA	➀	CCMD-2-R
		G2	10	NA	26.0+3.1	NA	TCM+MMT	/				
Zhang et al. (34)	2	G1	56	NA	28.6 ± 7.0	12.46 ± 18.64 m	EA+MMT	SJ5, PC6, PC8, LI4	19	NA	➀	CCMD-2-R
		G2	56	NA	28.6 ± 7.0	12.46 ± 18.64 m	MMT	/				

*CCMD-2-R, Chinese Classification of Mental Disorders, revised second edition; DSM-III-R, Diagnostic and Statistical Manual of Mental Disorders, revised third edition; DSM-IV, Diagnostic and Statistical Manual of Mental Disorders, fourth edition; CCMD-III, Chinese Classification of Mental Disorders, third edition; MA, manual acupuncture; EA, electro-acupuncture; MMT, methadone maintenance treatment; AA, auricular acupuncture; TCM, traditional Chinese medicine; TEAS, transcutaneous electrical acupoint stimulation; NA, not available; y, year; m, month; d, day; ➀ effective rate; ➁ MHOWS*.

The drug-taking manner included snuffing, snorting, intramuscular injection, and intravenous injection. Heroin was the most common drug in all the trials. Interventions contained the stimulating acupoints, electric current, frequency and specification of the needles, and electric stimulation equipment. All studies reported the treatment duration and six of them reported the follow-up. The treatment duration ranged within 7–90 days. Seventeen studies reported the course of opioid dependence patients.

For convenience, the control groups that used methadone maintenance treatment (MMT) as an opioid-substitution were summarized as Western medicine (WM) groups. The Chinese medical formulae, Chinese formulated products, and Chinese herbal detoxification capsules were summarized as traditional Chinese medicine (TCM) groups. Since MMT is a basic treatment for opioid dependence, we omitted MMT in the acupuncture-related therapy groups.

Ultimately, 1,661 participants in 16 two-arm studies and 336 participants in 4 three-arm studies were included for the network meta-analysis. In the two-arm studies, 3 of 16 trials compared manual acupuncture (MA) and Western medicine (WM) (15, 16, 27), 4 trials compared auricular acupuncture (AA) and WM (18, 19, 24, 25), 7 trials compared electro-acupuncture (EA) and WM (20–22, 26, 28, 31, 34), and 2 trials compared transcutaneous electrical acupoint stimulation (TEAS) vs. traditional Chinese medicine (TCM) (32), and EA vs. TCM (33), respectively. These three-arm studies performed comparisons among MA, WM and TCM (17), MA, EA and WM (23), EA, TCM and WM (29), and MA, WM and TEAS (30), respectively.

**Figure 2:**
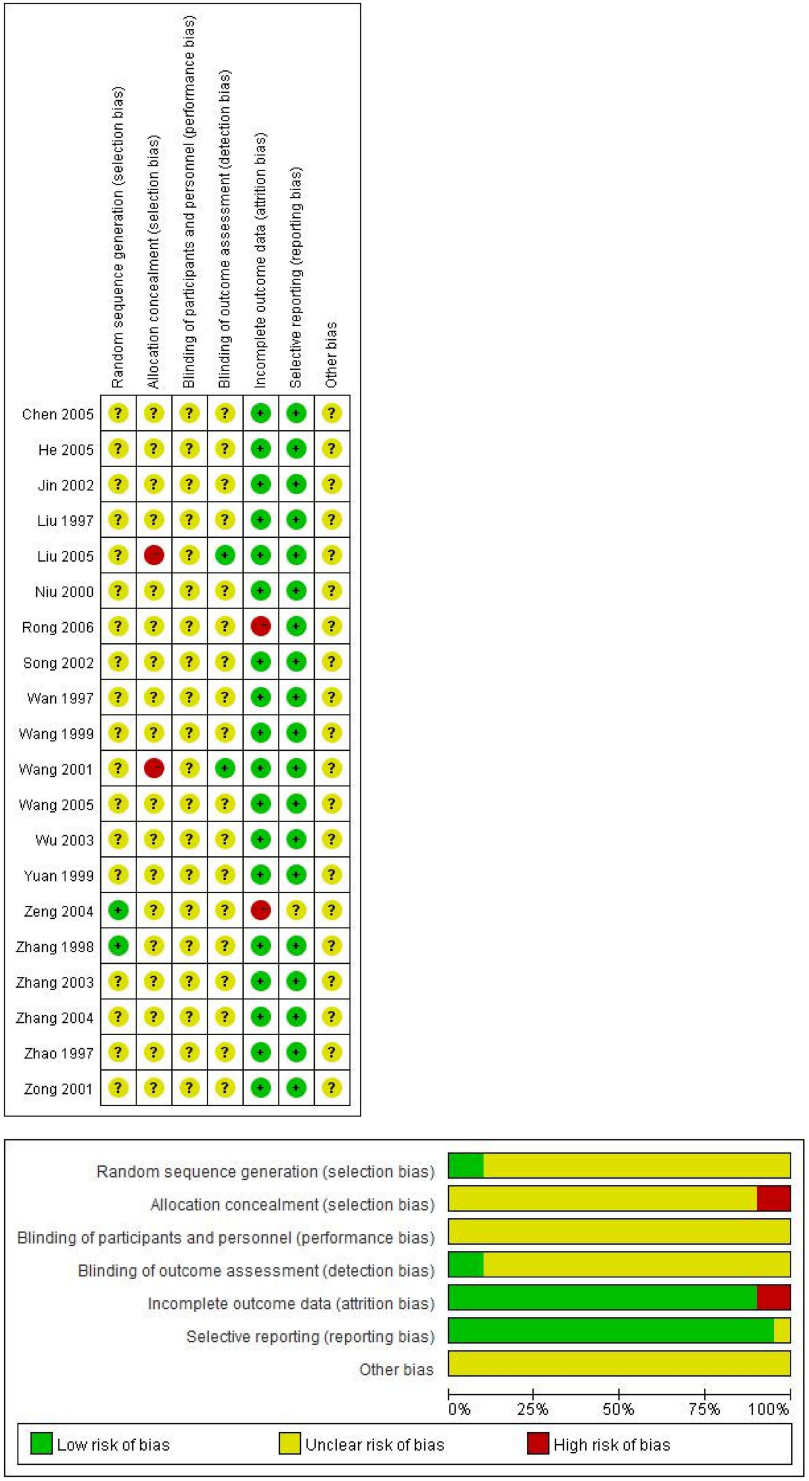
Risk of bias graph and summary.

For the outcome measures, 11 trials reported the effective rate (15, 18, 19, 22, 24–26, 31–34), 6 trials reported the MHOWS score (16, 20, 21, 23, 29, 30), and 3 trials reported both (17, 27, 28). Five of these selected trials reported their funding resources (15, 21, 22, 29, 30), while the remaining trials did not. Due to Cochrane's risk of bias tool, details shown in Figure 2, no trials were at low risk of bias.

### Meta-Analysis

In order to measure the efficacy of these six interventions, a classic meta-analysis was performed using the random-effects model, in order to compound studies with the same interventions. We considered a presence of statistically significant difference by setting the *P*-value to < 0.05. In order to analyze the effect sizes, risk ratios (RRs) were used for the effective rate and mean difference (MD) was used for the MHOWS score. The forest plots are shown in the Supplementary Files(Supplementary Figures 1, 2).

#### Primary Outcome: Effective Rate

A total of 14 studies that covered 8 head-to-head comparisons reported the effective rate (Supplementary Figure 1). According to the RRs and 95% CIs for effective rate, MA (1.27, 95% CI: 0.96 to 1.68, *P* = 0.34), AA (1.10, 95% CI: 0.93 to 1.31, *P* < 0.01), EA (1.04, 95% CI: 1.00 to 1.08, *P* = 0.05), and TCM (1.37, 95% CI: 0.98 to 1.92) had a higher effective rate, when compared to WM. Furthermore, TEAS (0.90, 95% CI: 0.77 to 1.05) was less efficacious than WM. For the comparisons among the other four acupuncture-related therapies, only one study compared and reported the data (TEAS vs. TCM: 1.36, 95% CI: 0.82 to 2.27; MA vs. TCM: 1.16, 95% CI: 0.96 to 1.41; EA vs. TCM: 0.80, 95% CI: 0.35 to 1.82). However, no statistical difference was found in the above comparisons.

#### Secondary Outcome: MHOWS

For the MHOWS, we judged lower scores of MHOWS as less pertinent symptoms of participants. Nine studies that covered eight direct comparisons reported the effects of five interventions (Supplementary Figure 2).

A significant decrease in MHOWS score was observed in MA comparing to WM (−8.59, 95% CI: −15.96 to −1.23, *P* < 0.01). TCM (−2.39, 95% CI: −3.66 to −1.13], *P* = 0.41), EA (−3.92, 95% CI: −9.01 to 1.17, *P* < 0.01), and TEAS (−4.23, 95% CI: −11.36 to 2.90) were better in decreasing the MHOWS score when compared to WM; however, no statistical differences were found in the comparisons. For the direct comparisons among acupuncture-related therapies, MA was more efficacious than EA (−6.15, 95% CI: −9.45 to −2.85) and TEAS (−10.44, 95% CI: −16.11 to −4.77). In comparing with TCM, EA (−0.45, 95% CI: −2.57 to 1.67) and MA (−0.72, 95% CI: −2.12 to 0.68) had better effects on the MHOWS score, although no statistical difference was found in the above comparisons.

### Cumulative Meta-Analysis

We conducted the cumulative meta-analysis by updating the pooled estimate of the studies according to the published year. The effective rate in comparisons of EA vs. WM (RR: 1.04, 95% CI: 1.00 to 1.08), AA vs. WM (RR: 1.10, 95% CI: 0.94 to 1.27), and MA vs. WM (1.27, 95% CI: 0.96 to 1.68), and the MHOWS in comparisons of EA vs. WM (MD: −3.92, 95% CI: −9.01 to 1.17), MA vs. WM (MD: −8.59, 95% CI: −15.96 to −1.23), and TCM vs. WM (MD: −2.39, 95% CI: −3.66 to −1.13) were supportive of the results in meta-analyses. The forest plots were in the Supplementary File (see in Supplementary Figures 3, 4).

### Network Meta-Analysis

**Figure 3:**
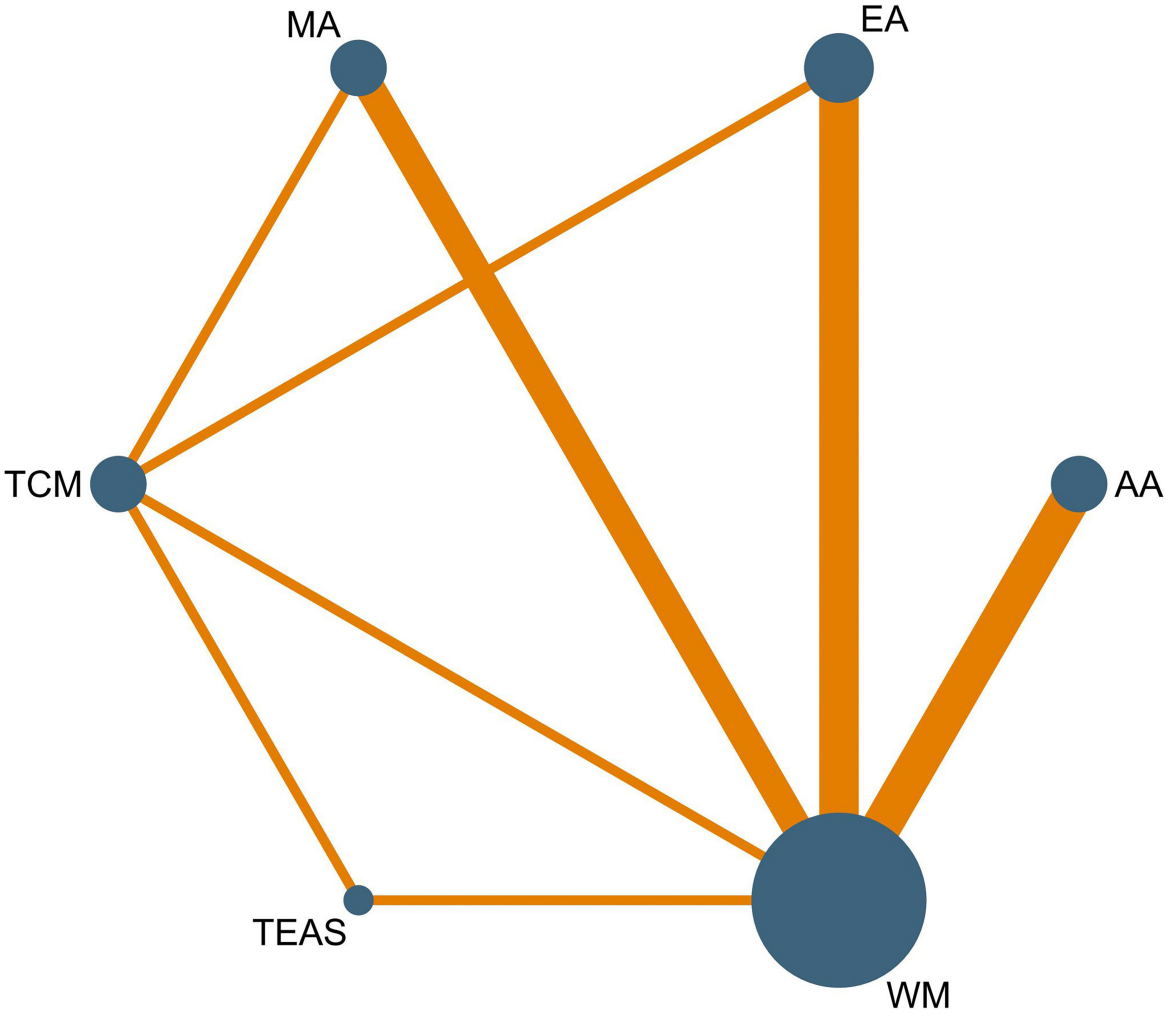
Network plot of effective rate.

**Figure 4:**
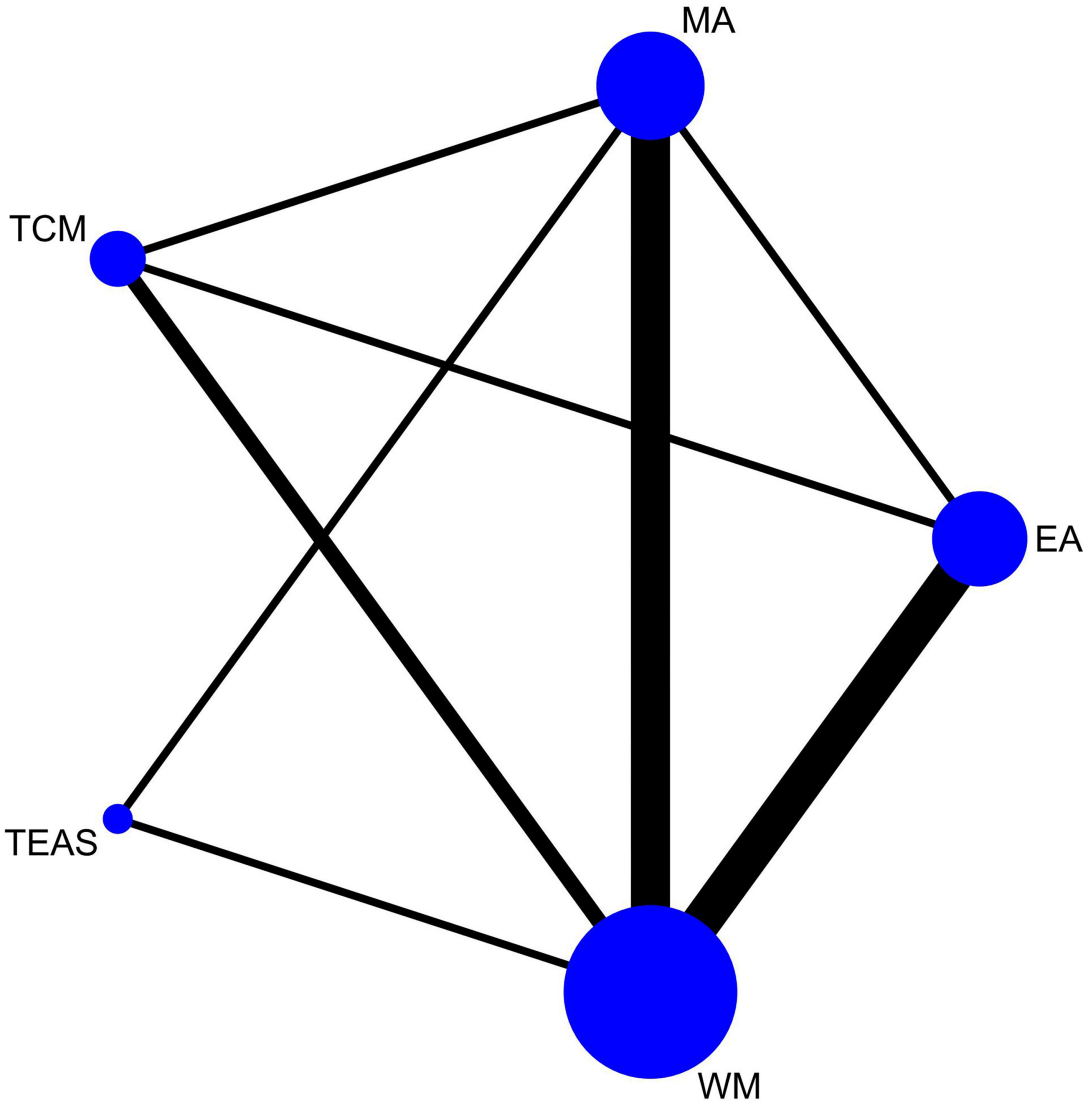
Network plot of MHOWS.

We conducted a network meta-analysis to exhaustively compare and rank the different interventions for opioid dependence. The network plots for the effective rate and MHOWS are presented in Figures 3, 4. Each spot represents an intervention, and the magnitude of the spot corresponds to the number of studies that contained the intervention. The width of the line between these two intervention spots indicates the quantity of the comparisons. In the network plots, all interventions had at least one comparison to WM.

#### Primary Outcome: Effective Rate

We used node-splitting analysis to assess the consistency, and all *P*-values between the direct and indirect effects were >0.05. The PSRF value close to 1 indicated that the convergence of the model was more suitable, and that the result was stable and convincing.

**Figure 5:**
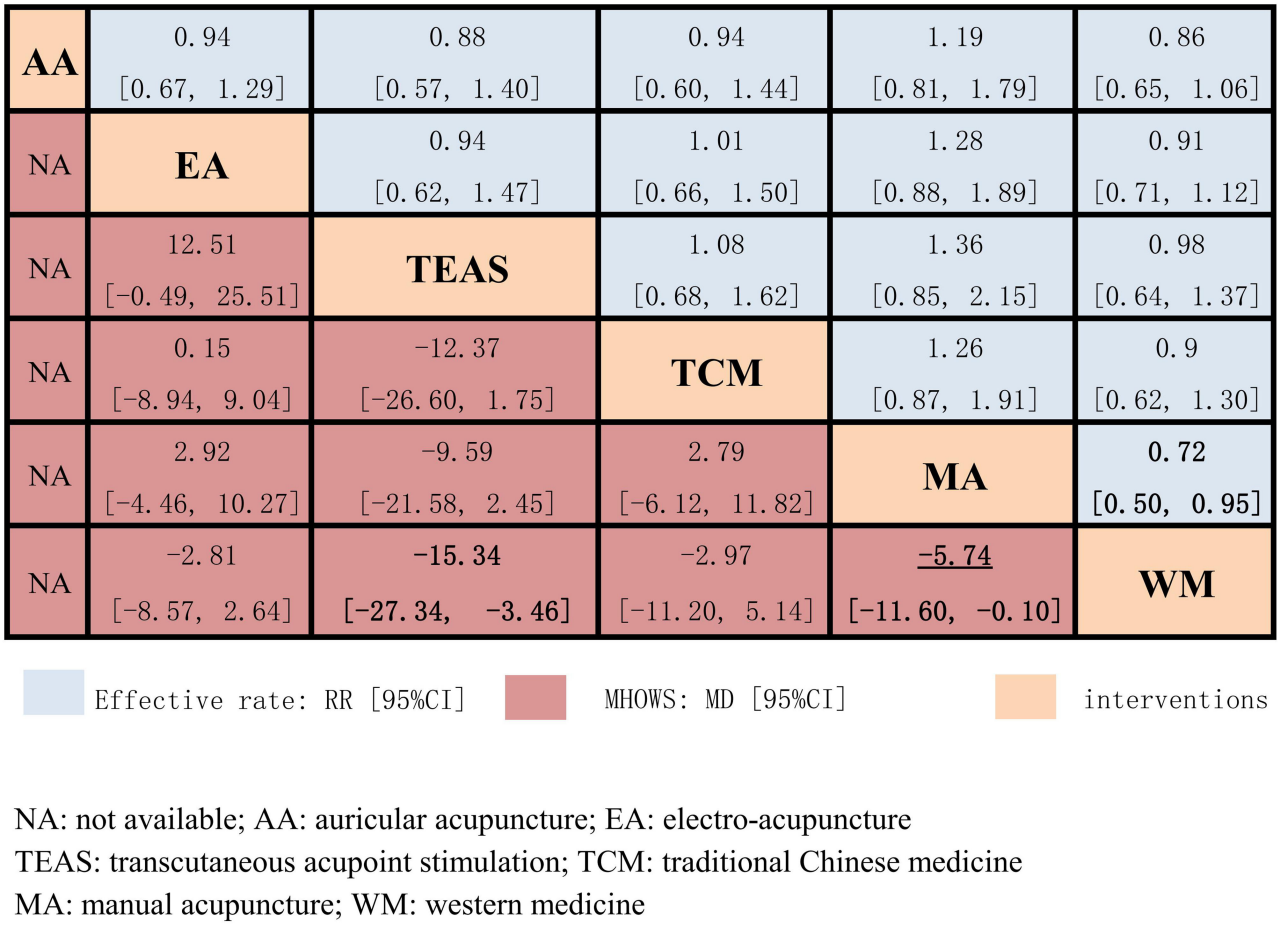
Effective rate and MHOWS of the 6 interventions.

**Figure 6:**
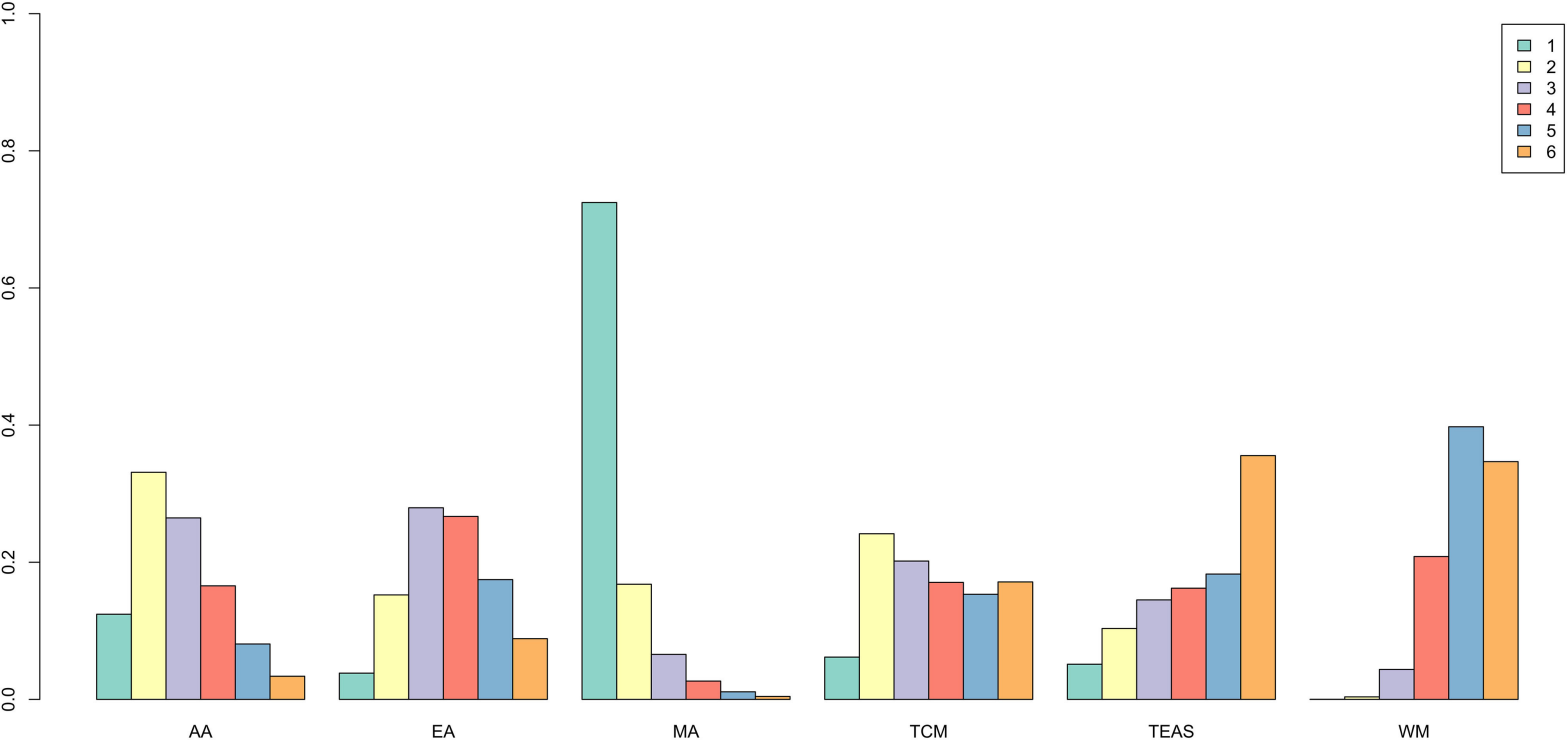
Rank probability of effective rate.

The network meta-analysis for the effective rate is presented in Figure 5. In the comparisons, WM was the least efficacious among all interventions (MA: 0.72, 95% CI: 0.50 to 0.95; TEAS: 0.98, 95% CI: 0.64 to 1.37; EA: 0.91, 95% CI: 0.71 to 1.12; AA: 0.86, 95% CI: 0.65 to 1.06). However, a statistically significant difference was only observed in MA vs. WM. The ranking probability of the effective rate (Figure 6 and Table 2) strongly indicated that MA had the highest probability (88.8%) to be the best treatment for opioid dependence, followed by AA (3.7%), EA (3.4%), TEAS (2.1%), TCM (2%), and WM (0.01%). Another supportive evidence from the SUCRA plot (Supplementary Figure 11) revealed that MA has the maximum area under the curve. Hence, MA was the most efficacious intervention for opioid dependence.

**Table 2:** Rank probability of effective rate.

**Rank**	**AA**	**EA**	**MA**	**TCM**	**TEAS**	**WM**
1	0.037	0.034	0.888	0.020	0.021	0.0001
2	0.241	0.321	0.064	0.236	0.135	0.004
3	0.201	0.307	0.026	0.260	0.163	0.043
4	0.189	0.207	0.013	0.222	0.193	0.176
5	0.177	0.101	0.006	0.159	0.192	0.365
6	0.156	0.031	0.003	0.103	0.295	0.411

#### Secondary Outcome: MHOWS

**Figure 7:**
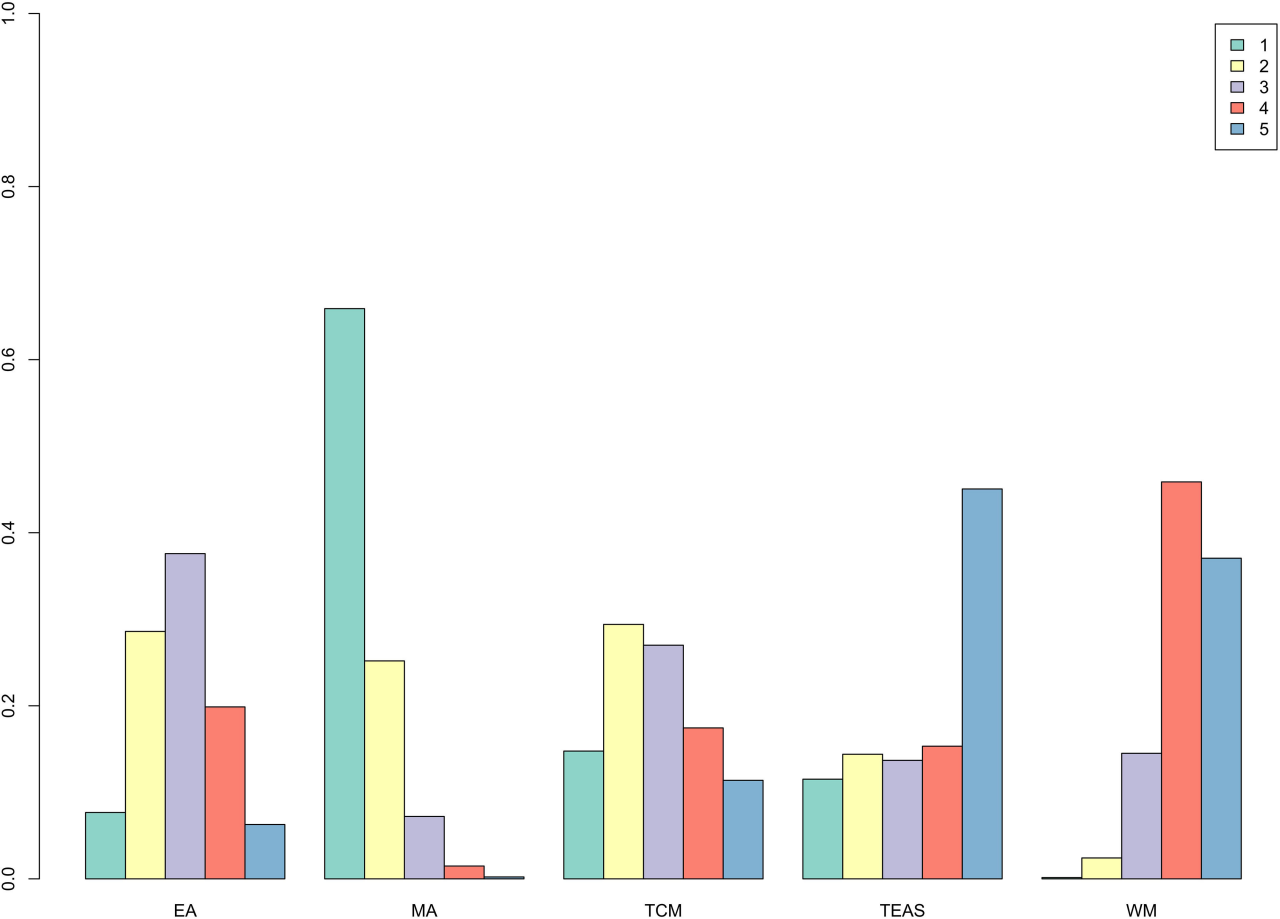
Rank probability of MHOWS.

In the results of the network meta-analysis for the MHOWS (Figure 5), all acupuncture-related therapies exhibited superior effects, when compared to Western medicine (MA: −5.74, 95% CI: −11.60 to −0.10; TEAS: −15.34, 95% CI: −27.34 to −3.46; EA: −2.81, 95% CI: −8.57 to 2.64). Significant differences were observed in TEAS vs. WM, and MA vs. WM. In relieving withdrawal symptoms, MA had the highest probability (65.6%) to rank first among the five interventions (Figure 7 and Table 3), followed by TCM (15%), TEAS (11.6%), EA (7.7%), and WM (0.1%). The SUCRA plot (Supplementary Figure 12) also demonstrated that manual acupuncture was the highest-ranked intervention in MHOWS reduction, while WM had the lowest SUCRA.

**Table 3:** Rank probability of MHOWS.

**Rank**	**EA**	**MA**	**TCM**	**TEAS**	**WM**
1	0.077	0.656	0.150	0.116	0.001
2	0.288	0.253	0.291	0.144	0.024
3	0.376	0.073	0.271	0.137	0.144
4	0.196	0.016	0.173	0.153	0.462
5	0.063	0.002	0.115	0.450	0.369

### Inconsistency and Heterogeneity Check

Node-split was conducted to detect the inconsistency and heterogeneity for the effective rate and MHOWS score (Supplementary Figures 9, 10). The direct evidence approximately conformed with the indirect evidence due to the *P*-value of > 0.05. The node-split plots revealed that there was barely a heterogeneity or inconsistency between the direct and indirect evidence in each comparison.

## Discussion

To date, the present study is the first to conduct a network meta-analysis on various therapies for opioid dependence patients receiving MMT. This network meta-analysis would help to build up connections between individual interventions, evaluate the efficacy of all therapies, and conclude the best treatment by ranking these therapies. From the results of the present network meta-analysis and the rank of the interventions, and among all the acupuncture-related therapies, manual acupuncture has a prominent effect on the effective rate and MHOWS score. Furthermore, both the direct evidence in the meta-analysis and indirect evidence made by Bayesian statistical methods indicated that manual acupuncture may effectively allay the withdrawal symptoms, and help patients get rid of opioid dependence. Some studies (37) demonstrated that opiate withdrawal symptoms are relevant to the endorphins, enkephalins, and dynorphins from the brain. Hence, stimulating some acupoints would induce the brain to release these substances, thereby relieving the pain from opiate withdrawal. It is reasonable that acupuncture can trigger many endogenous opioids inside the body, while cutting off the exogenous addictive substances. With this painless detoxification, patients can stop taking medications when the endogenous opioid secretion reaches the normal level.

At present, many patients desire to completely throw off addiction and live in a normal way without taking methadone. However, for the patients who are used to their daily dosage, it is difficult to change their dosage. To avoid withdrawal syndrome caused by reducing methadone, most of them choose to take the same dosage, even for the rest of their lives. The most reason we highly recommend acupuncture for MMT patients is that acupuncture, as the adjunctive therapy, can largely improve the phenomenon, helping patients get rid of opioids by controlling withdrawal syndrome. Acupuncture is now being used in many diseases (38), the safety and efficacy have been certified for many years. Since long-term opioid therapy led to iatrogenic addiction (39), acupuncture is highly worth recommending for relieving the psychological burden of patients without euphoric effects or risk of addiction. According to these present results, acupuncture plus methadone is more effective than methadone only, which indicates that MMT industries can provide acupuncture treatment for opioid dependence patients, in order to achieve better compliance and detoxification.

Compared to the former meta-analysis, one of which focused on the effect of acupuncture combined with opioid receptor agonists in treating opiate-withdrawal symptoms, the present study included and reported in detail the different types of acupuncture and ranked the possible best intervention for opioid dependence. Another familiar direction is the comparison of the dose response and efficacy among opiate maintenance treatment, burenorphine, and other pharmacological adjunctive interventions (40–43). The objective of the present study is to prove the effectiveness and feasibility of acupuncture-related therapies in treating opioid dependence patients receiving MMT.

Although the present network meta-analysis strongly persuades that acupuncture-related therapies are more effective than WM, there were some limitations of the present study. First, the RCTs were published at least 15 years ago which commonly had no rigorous study design, or a detailed description on randomization, allocation concealment, or blinding. Therefore, the low quality of the included trials may downgrade the confidence on the recommendation. Second, the two outcome measures were both correlated with the MHOWS score, which indicates that the efficacy was only judged by the decreasing scores. In order to determine the independent effect of different types of acupuncture therapies in the future, the daily reduction of methadone while receiving acupuncture combined with MMT can be measured, which may more directly contribute to a positive effect in opioid dependence. Last, the relapse of patients varied from person to person, that is, some of the patients were at their first abstinence, while some of the patients may have undergone this twice or had more relapses. This may affect the present results in a way. If possible, researchers can collect more information and explore the interior connection between relapse and detoxification.

## Conclusion

The existing evidence shows that acupuncture related-therapies may effectively be used for treating patients receiving MMT, and the results of this network meta-analysis support manual acupuncture may be the best choice for opioid dependence among all kinds of acupuncture-related therapies. Nevertheless, reducing the relapse and promoting the recovery of opioid dependence needs more efforts from not only the medical industry but also government support, security system, and educational popularization. To strengthen the assurance of acupuncture-related therapies in the treatment of opioid dependence, we expected that clinical trials with high quality would be conducted, to provide more confident evidence.

## Data Availability Statement

The original contributions presented in the study are included in the article/Supplementary Material, further inquiries can be directed to the corresponding author/s.

## Author Contributions

HW and LL designed the search strategy. SG and WL performed the literature search. HW and RC screened the studies for eligibility and wrote the first draft of the manuscript. RC, YD, and XW performed the data extractions. HW, RC, and PZ conducted the statistical analyses. LL, SX, and YZ were responsible for the manuscript editing and review of the manuscript.

## Funding

This work was supported by the National Natural Science Foundation of China (82174527), the special project of Lingnan modernization of traditional Chinese medicine in 2019 Guangdong Provincial R & D Program (2020B1111100008), the High-level university construction of GZUCM (A1-2601-21-415-024), the National Natural Science Foundation of China (81872261 to SX); the Scientific Project of Guangzhou (201803010013 to SX), and the Luo Yongjia Famous Traditional Chinese Medicine Studio, Guangdong Traditional Chinese Medicine Office ([2019] No. 5 to YZ).

## Conflict of Interest

The authors declare that the research was conducted in the absence of any commercial or financial relationships that could be construed as a potential conflict of interest.

## Publisher's Note

All claims expressed in this article are solely those of the authors and do not necessarily represent those of their affiliated organizations, or those of the publisher, the editors and the reviewers. Any product that may be evaluated in this article, or claim that may be made by its manufacturer, is not guaranteed or endorsed by the publisher.
