# Supplementary material for: Acupuncture for Opioid Dependence Patients Receiving Methadone Maintenance Treatment: A Network Meta-Analysis
Source: Front Psychiatry. 2021 Dec 13;12:767613. doi: 10.3389/fpsyt.2021.767613 (PMC8710762; doi:10.3389/fpsyt.2021.767613)
Supplement: Supplementary file 1 [file Data_Sheet_1.ZIP › Supplementary files/Supplementary file-Search terms.docx]

Appendix 1: Search terms and search strategies for each database

PubMed：

#1 Search (“acupuncture”[Title/Abstract] OR “electro-acupuncture” [Title/Abstract] OR “warming needling” [Title/Abstract] OR “fire needling”[Title/Abstract] OR “bloodletting”[Title/Abstract] OR

“auriculo-acupuncture”[Title/Abstract] OR “auricular”[Title/Abstract] OR “moxibustion”[Title/Abstract]OR, “cupping” [Title/Abstract]OR “acupoint*” [Title/Abstract])

#2 Search (“heroin”[Title/Abstract] OR “withdrawal syndrome”[Title/Abstract] OR “opioid”[Title/ Abstract] OR “opioid dependence”[Title/Abstract] OR “addiction” [Title/Abstract] OR “drug abuse”[Title/Abstract] OR “substance use disorder” [Title/Abstract] OR “substance use disorder” [Title/Abstract]OR “methadone” [Title/Abstract])

#3 Search (“randomized controlled trial” [Title/Abstract] OR “controlled clinical “[Title/Abstract] OR “trial “[Title/Abstract] OR “group”[Title/Abstract] OR “placebo” [Title/Abstract] OR “randomly” [Title/Abstract])

#4 Search (#2 and #3)

#5 Search (#4 and #5)

Medline Database：

1 exp Heroin/

2 exp Methadone/

3 exp Drug addicts/

4 exp Opioid dependence/

5 exp Opioid use disorder/

6 exp Substance use disorder/

7((Withdrawal) adj3 (syndrome or symptom*)). ti,ab.

8 or/1-7

9 exp Acupuncture/

10 exp Acupuncture Therapy/

11 (acupuncture or acupoint* or meridian*). ti,ab.

12 (electroacupuncture or electro-acupuncture). ti,ab.

13 (acupressure* or acup* point* or mox* or needl* or

auriculo-acup* or cup* or bloodlet*). ti,ab.

14 or/9-13

15 8 and 14

16 randomized controlled trial. ti,ab.

17 controlled clinical trial.ti,ab.

18 randomi$.ti,ab.

19 randomly.ti,ab.

20 trial.ti,ab.

21 group.ti,ab.

Embase database：

1. exp Heroin/

2. exp Methadone/

3. exp Drug addicts/

4. exp Opioid dependence/

5. exp Opioid use disorder/

6((Withdrawal) adj3 (syndrome or symptom*)). ti,ab.

7 or/1-6

8 exp Acupuncture/

9 exp Acupuncture Therapy/

10 (acupuncture or acupoint* or meridian*). ti,ab.

11 (electroacupuncture or electro-acupuncture). ti,ab.

12 (acupressure* or acup* point* or mox* or needl* or auriculo-acup* or cup* or bloodlet*). ti,ab.

13 or/8-12

14 7 and 13

15 randomized controlled trial. ti,ab.

16 controlled clinical trial.ti,ab.

17 randomi$.ti,ab.

18 randomly.ti,ab.

19 trial.ti,ab.

20 group.ti,ab.

21 placebo.ti,ab.

Cochrane Controlled Trials Register/CENTRAL in Cochrane Library：

#1 MeSH descriptor: [Withdrawal syndrome] explode all trees

#2 MeSH descriptor: [Opioid dependence] explode all trees

#3 MeSH descriptor: [Drug addicts] explode all trees

#4 MeSH descriptor: [substance abuse] explode all trees

#5 MeSH descriptor: [Methadone] explode all trees

#6 MeSH descriptor: [Heroin] explode all trees

#7 #1 OR #2 OR #3 OR #4 OR #5 OR #6

#8 MeSH descriptor: [Acupuncture] explode all trees

#9 MeSH descriptor: [Acupressure] explode all trees

#10 MeSH descriptor: [Moxibustion] explode all trees

#11 MeSH descriptor: [Bloodletting] explode all trees

#12 (acupuncture OR acupoint* OR meridian*):ti,ab,kw

#13 (electroacupuncture OR electro-acupuncture):ti,ab,kw

#14 (acupressure* OR acup* point* OR mox* OR needl* OR auriculo-acup* OR cup* OR bloodlet*):ti,ab,kw

#15 #8 OR #9 OR #10 OR #11 OR #12 OR #13 OR #14

#16 #7 AND #15

#17 MeSH descriptor: [Randomized Controlled Trial] explode all trees

#18 MeSH descriptor: [Clinical Trial] explode all trees

#19 (randomized controlled trial OR controlled clinical trial OR random* OR trial OR group):ti,ab,kw

#20 (placebo):ti,ab,kw

SCOPUS：

## ( acupuncture  OR  electro-acupuncture  OR  auricular  AND acupuncture  OR  warming  AND needling  OR  moxibustion )  AND  ( heroin  OR  addiction  OR  opioid  AND dependence  OR  opioid  AND use  AND disorder  OR  drug  AND abuse  OR  substance  AND use  AND disorder  OR  methadone )  AND  ( randomized  AND controlled  AND trial  OR  controlled  AND clinical )

Web of Science：

(( acupuncture OR electro-acupuncture OR auricular AND acupuncture OR warming AND needling OR moxibustion ) AND ( heroin OR addiction OR opioid AND dependence OR opioid AND use AND disorder OR drug AND abuse OR substance AND use AND disorder OR methadone ) AND ( randomized AND controlled AND trial OR controlled AND clinical ))

CNKI：

((SU='海洛因' OR SU='美沙酮' OR SU='依赖' OR SU='成瘾' OR SU='戒毒' OR SU='毒品' OR SU='阿片类药物依赖' OR SU='物质依赖 ') OR (SU='戒断症状' OR SU='戒断综合征' OR SU='物质使用障碍' OR SU='物质戒断综合征'))AND (SU='针刺'OR SU='针灸' OR SU='灸' OR SU='针法' OR SU='刺法' OR SU='体针' OR SU='腹针' OR SU='头针' OR SU='温针' OR SU='火针' OR SU='电针' OR SU='梅花针' OR SU='刺络' OR SU='放血' OR SU='拔罐' OR SU='耳穴' OR SU='穴位注射' OR SU='穴位按压' OR SU='穴位埋线') )AND (SU='试验' OR SU='随机' OR SU='对照'OR SU=‘临床研究’OR SU=‘临床观察’)

WanFang database：

((海洛因 + 美沙酮 + 依赖 + 成瘾 + 戒毒 + 毒品 + 阿片类药物依赖 + 物质依赖 ) + (题名或关键词:(戒断症状 + 戒断综合征 + 物质使用障碍 + 物质戒断综合征)) )* 题名或关键词: (针灸 + 针刺 + 灸 + 针法 + 刺法 + 体针 + 腹针 + 头针 + 温针 + 火针 + 电针 + 电磁针 + 梅花针 + 刺络 + 放血 + 拔罐 + 耳穴 + 穴位注射 + 穴位按压 + 穴位埋线) * 摘要: (试验 + 随机 + 对照 + 临床研究 +临床观察)

VIP database：

((M=(海洛因 + 美沙酮 + 依赖 + 成瘾 + 戒毒 + 毒品 + 阿片类药物依赖 + 物质依赖 ) + M =(戒断症状 + 戒断综合征 + 物质使用障碍 + 物质戒断综合征)) AND M =(针灸 + 针刺 + 灸 + 针法 + 刺法 + 体针 + 腹针 + 头针 + 温针 + 火针 + 电针 + 电磁针 + 梅花针 + 刺络 + 放血 + 拔罐 + 耳穴 + 穴位注射 + 穴位按压 + 穴位埋线)) AND R =(试验 + 随机 + 对照 + 临床研究 + 临床观察)
