# Supplementary material for: Acupuncture for Opioid Dependence Patients Receiving Methadone Maintenance Treatment: A Network Meta-Analysis
Source: Front Psychiatry. 2021 Dec 13;12:767613. doi: 10.3389/fpsyt.2021.767613 (PMC8710762; doi:10.3389/fpsyt.2021.767613)
Supplement: Supplementary file 1 [file Data_Sheet_1.ZIP › Supplementary files/Fig S6-1.Trace plot of MHOWS-1.pdf]

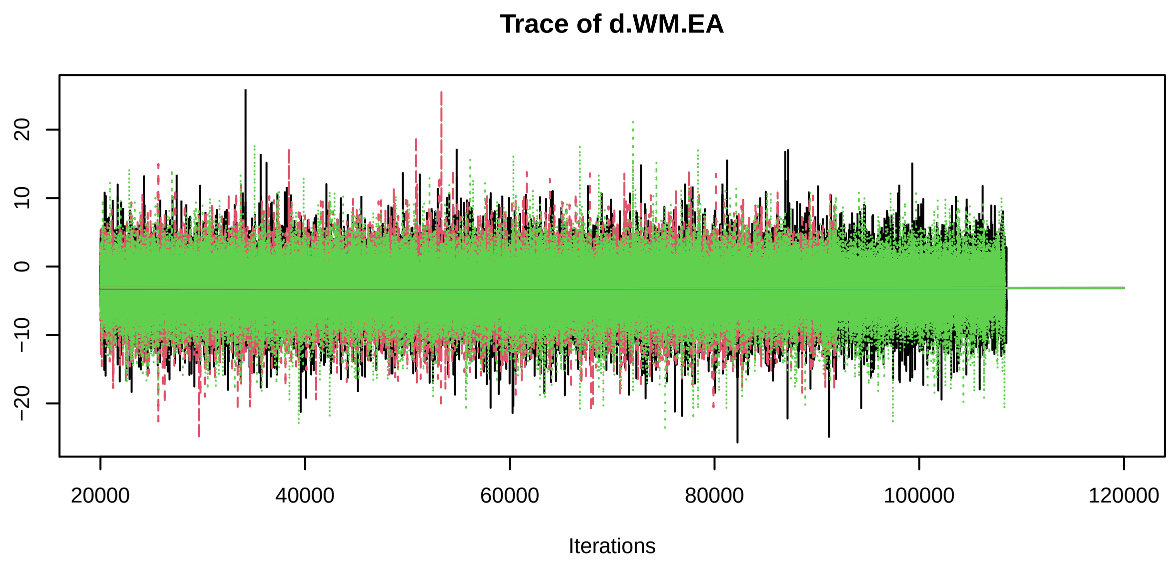
Trace of d.WM.EA
−20 −10 0 10 20
20000 40000 60000 80000 100000 120000
Iterations
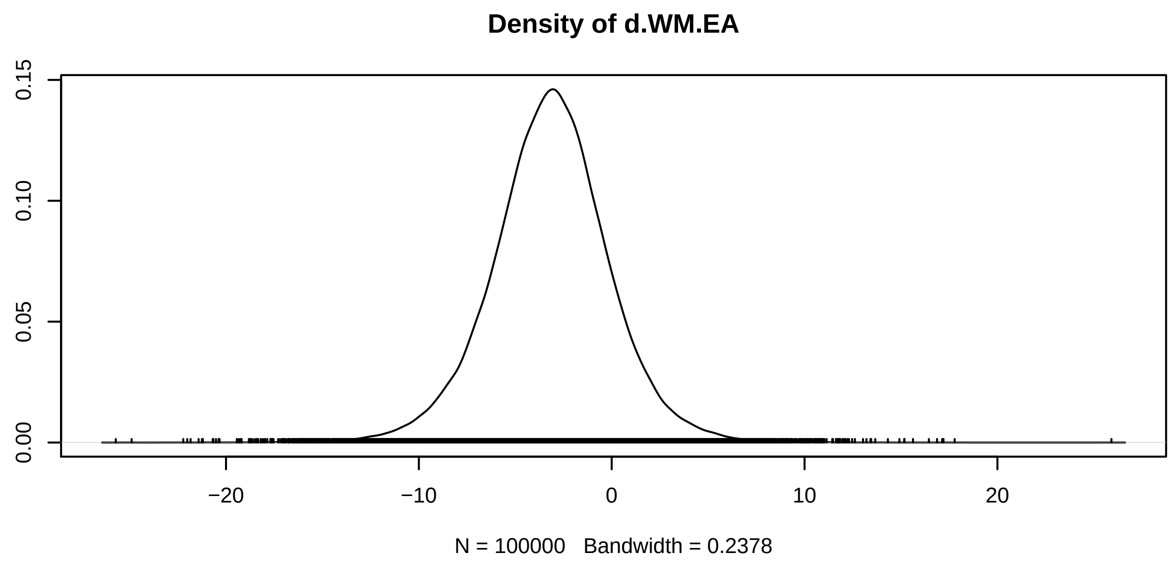
Density of d.WM.EA
0.00 0.05 0.10 0.15
−20 −10 0 10 20
N = 100000 Bandwidth = 0.2378
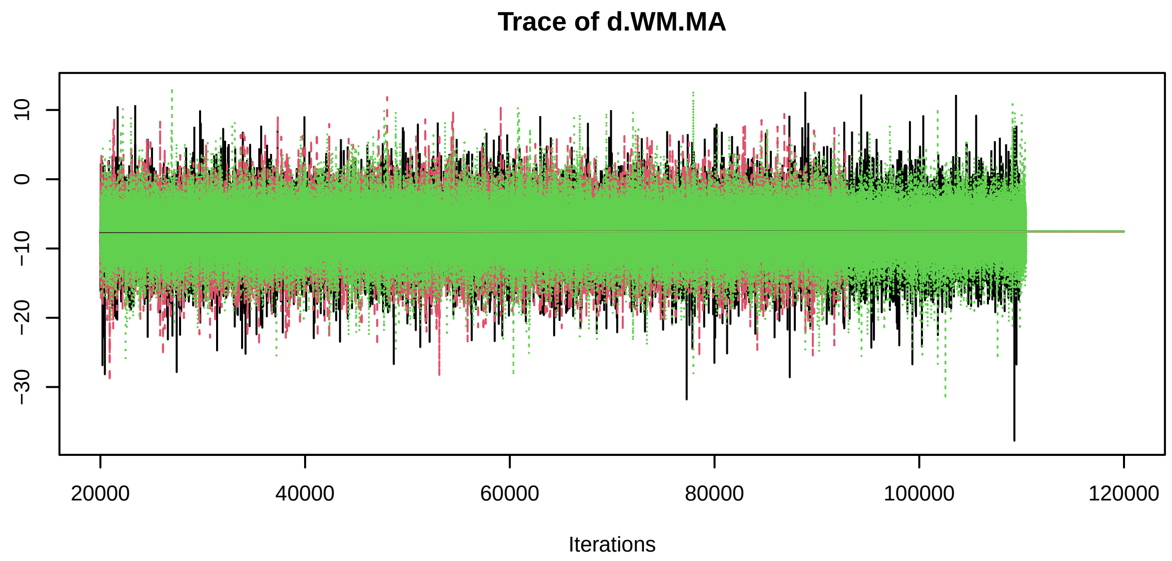
Trace of d.WM.MA
−30 −20 −10 0 10
20000 40000 60000 80000 100000 120000
Iterations
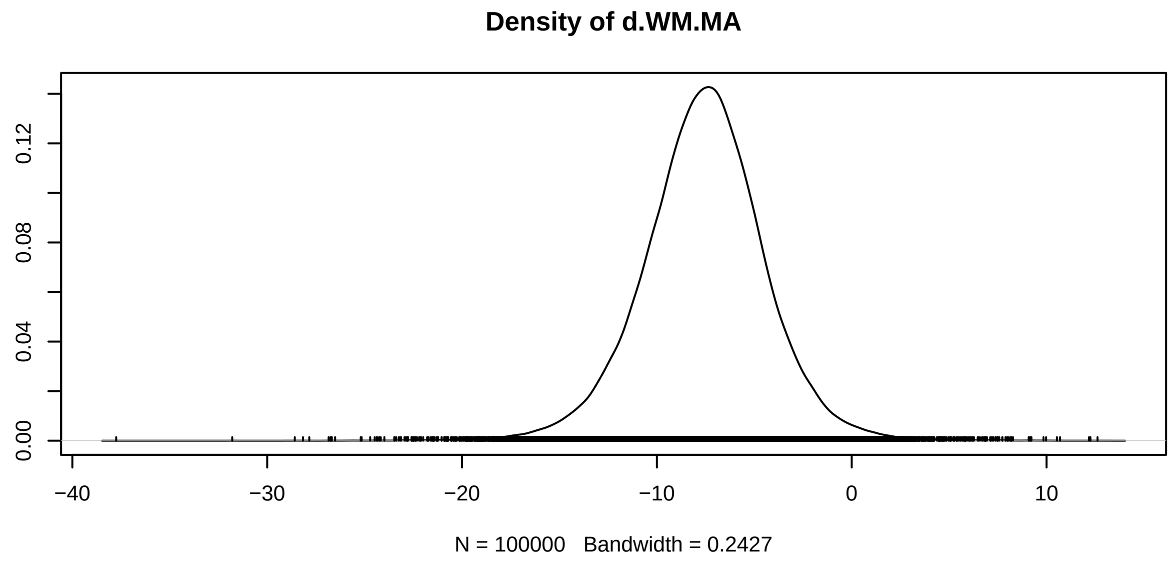
Density of d.WM.MA
0.00 0.04 0.08 0.12
−40 −30 −20 −10 0 10
N = 100000 Bandwidth = 0.2427
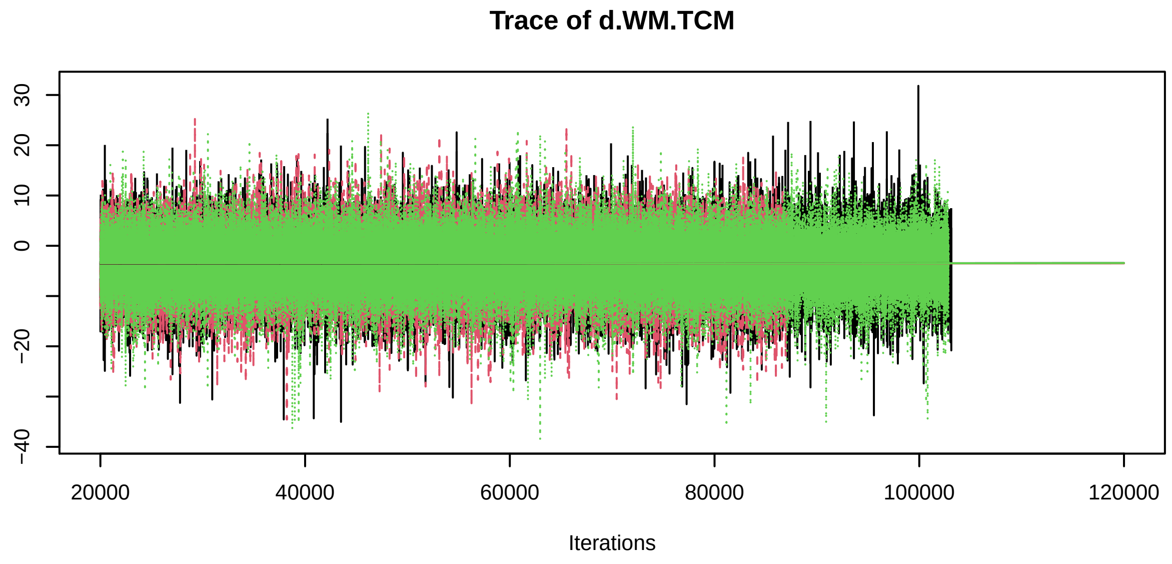
Trace of d.WM.TCM
−40 −20 0 10 20 30
20000 40000 60000 80000 100000 120000
Iterations
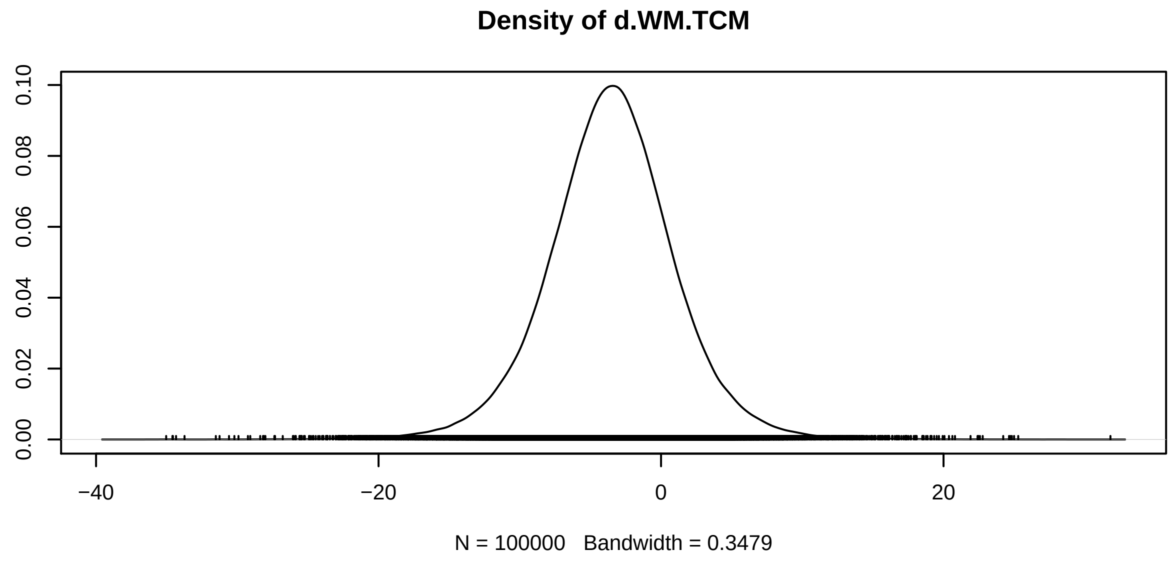
Density of d.WM.TCM
0.00 0.02 0.04 0.06 0.08 0.10
−40 −20 0 20
N = 100000 Bandwidth = 0.3479
