# Supplementary material for: Acupuncture for Opioid Dependence Patients Receiving Methadone Maintenance Treatment: A Network Meta-Analysis
Source: Front Psychiatry. 2021 Dec 13;12:767613. doi: 10.3389/fpsyt.2021.767613 (PMC8710762; doi:10.3389/fpsyt.2021.767613)
Supplement: Supplementary file 1 [file Data_Sheet_1.ZIP › Supplementary files/Fig S5-2.Trace plot of effective rate-2.pdf]

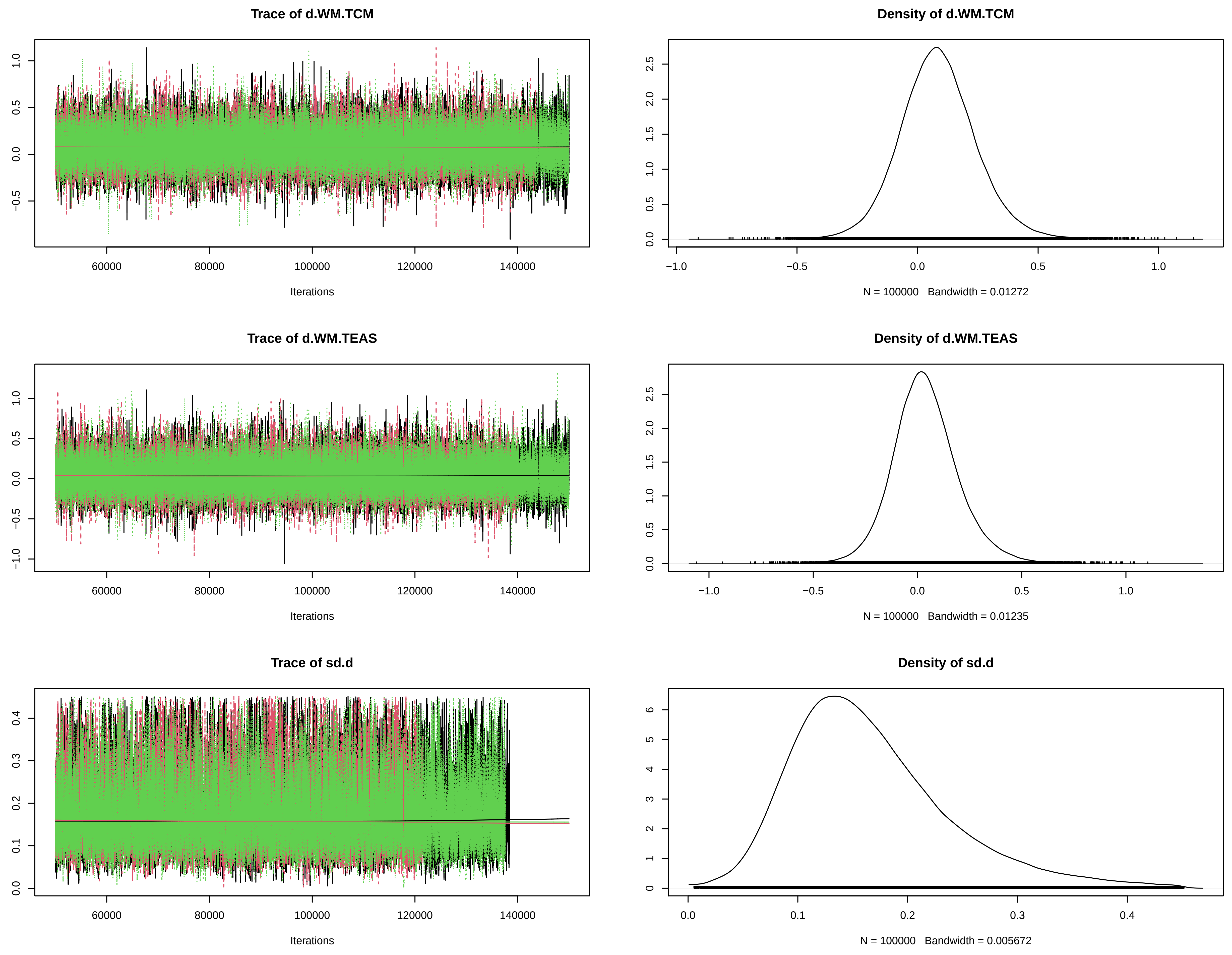
Trace of d.WM.TCM
Iterations
Density of d.WM.TCM
N = 100000 Bandwidth = 0.01272
Trace of d.WM.TEAS
Iterations
Density of d.WM.TEAS
N = 100000 Bandwidth = 0.01235
Trace of sd.d
Iterations
Density of sd.d
N = 100000 Bandwidth = 0.005672
