# Supplementary figures and images for: Acupuncture for Opioid Dependence Patients Receiving Methadone Maintenance Treatment: A Network Meta-Analysis
Source: Front Psychiatry. 2021 Dec 13;12:767613. doi: 10.3389/fpsyt.2021.767613 (PMC8710762; doi:10.3389/fpsyt.2021.767613)

**d.WM.EA**

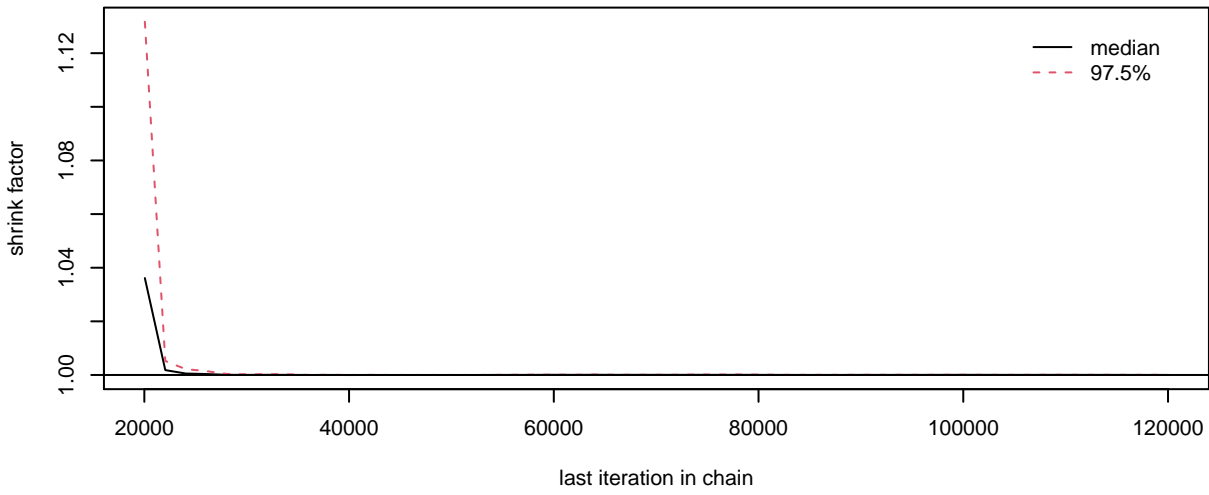

**d.WM.MA**

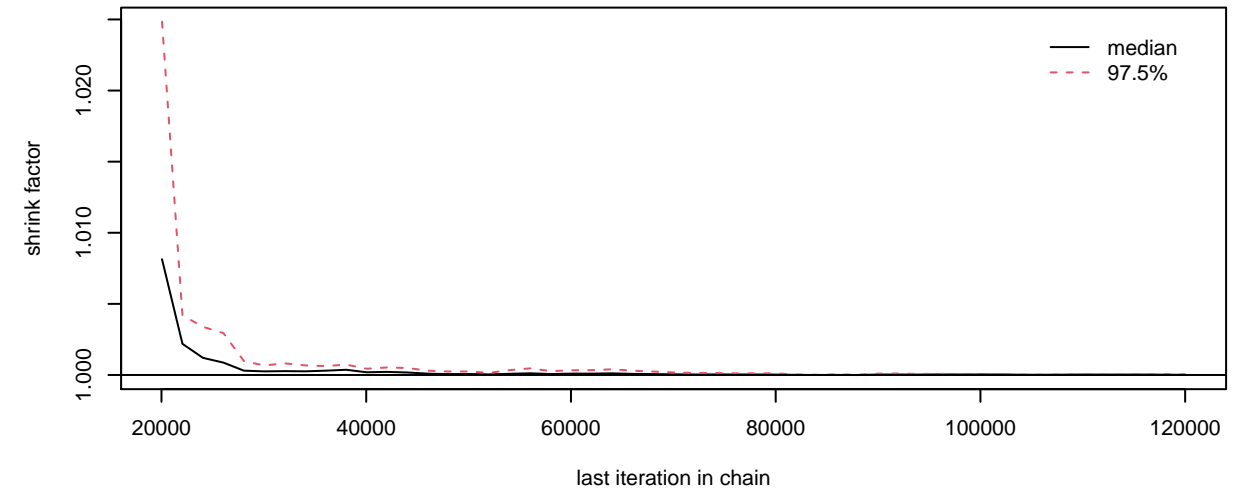

**d.WM.TCM**

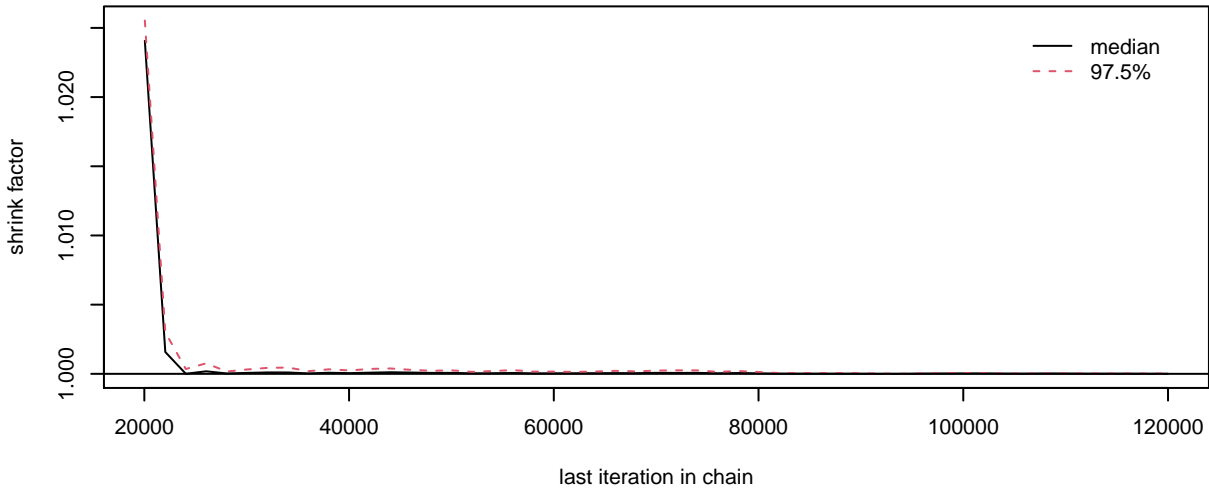

**d.WM.TEAS**

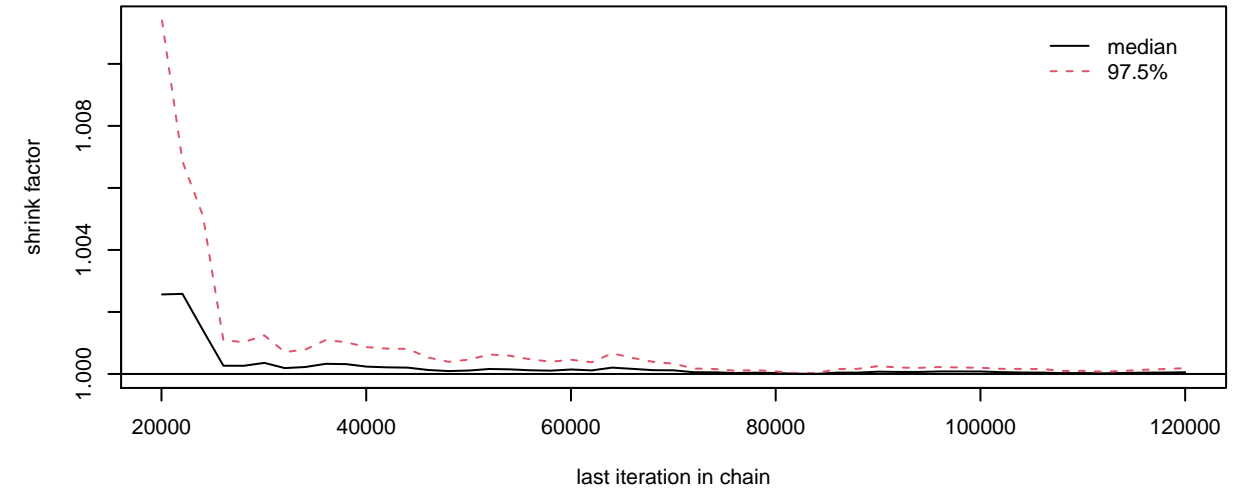

**sd.d**

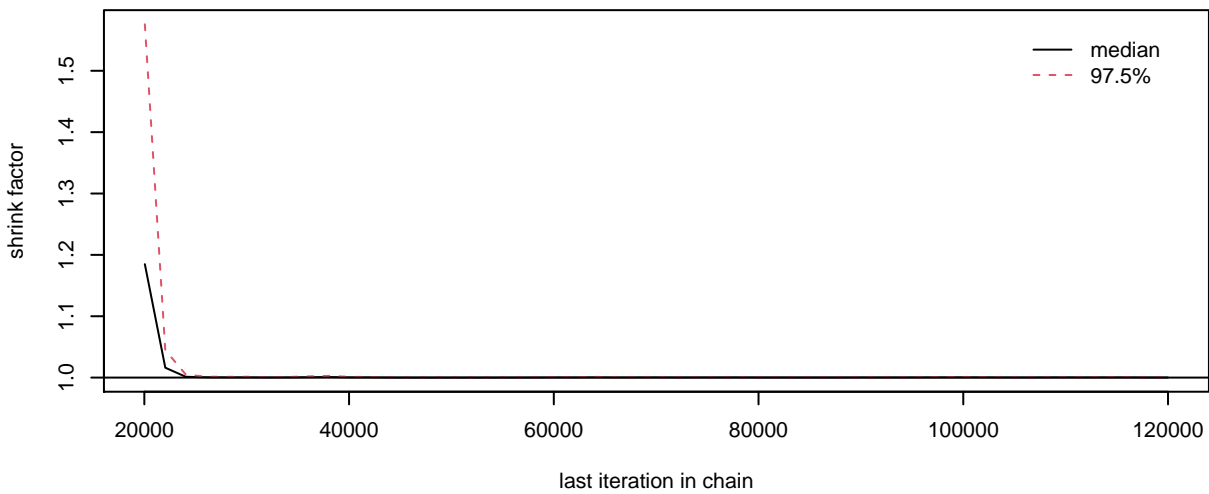

Supplement: Supplementary file 1 [file Data_Sheet_1.ZIP › Supplementary files/Fig S8. Gelman-Rubin-Brooks plot of MHOWS.pdf]

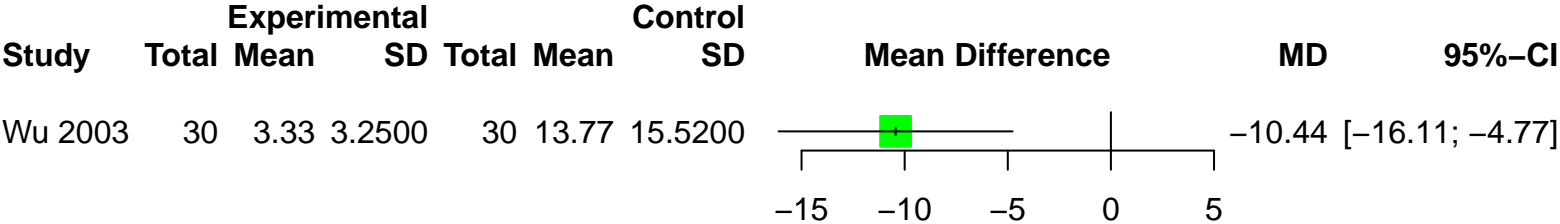

Supplement: Supplementary file 1 [file Data_Sheet_1.ZIP › Supplementary files/Fig S2-5.MHOWS.MAvsTEAS.pdf]

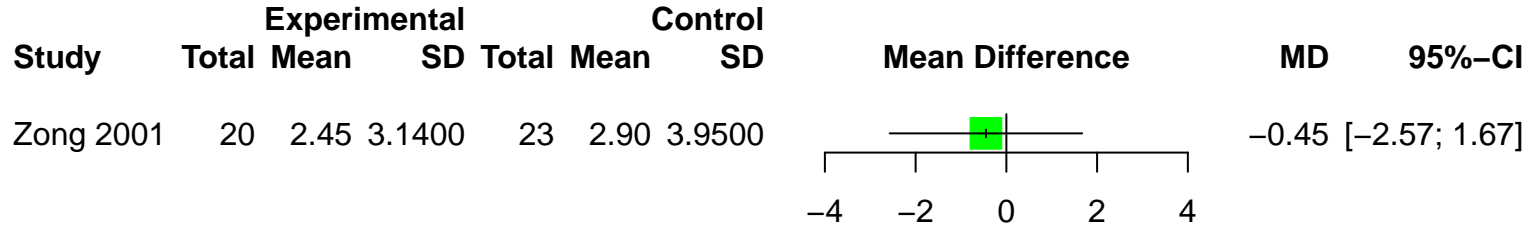

Supplement: Supplementary file 1 [file Data_Sheet_1.ZIP › Supplementary files/Fig S2-1.MHOWS.EAvsTCM.pdf]

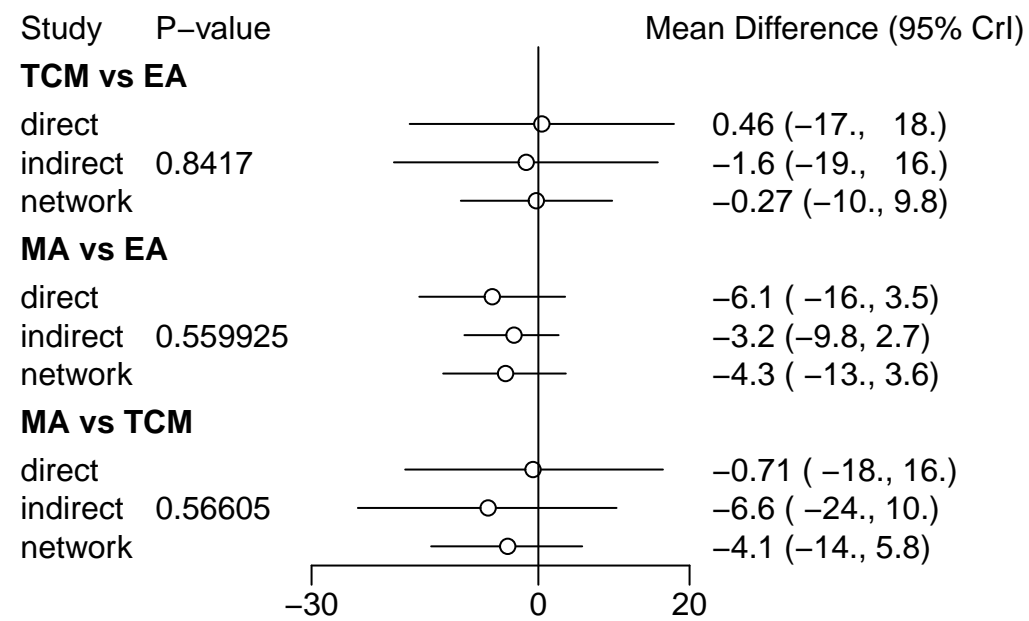

Supplement: Supplementary file 1 [file Data_Sheet_1.ZIP › Supplementary files/Fig S10. Node-splitting plot of MHOWS.pdf]

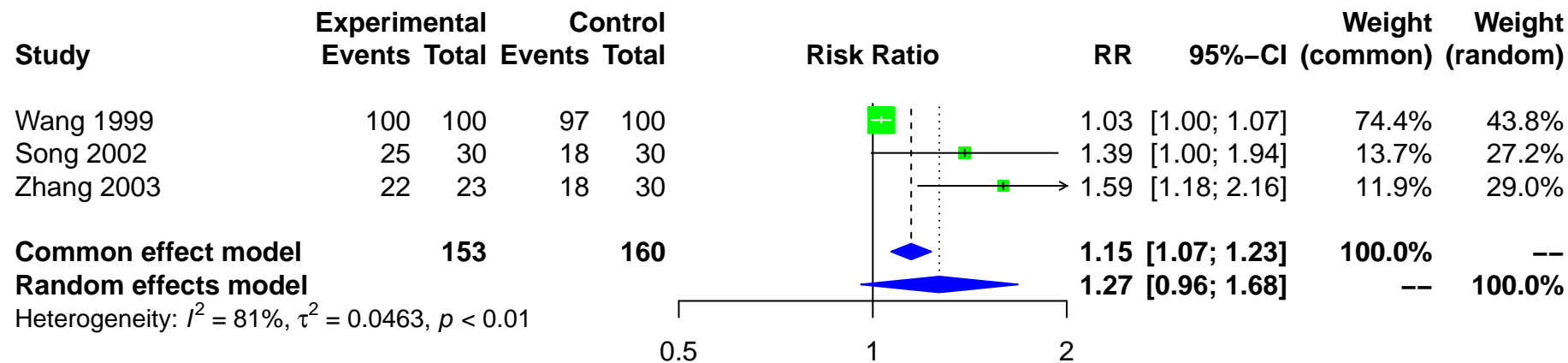

Supplement: Supplementary file 1 [file Data_Sheet_1.ZIP › Supplementary files/Fig S1-5.ER.MAvsWM.pdf]

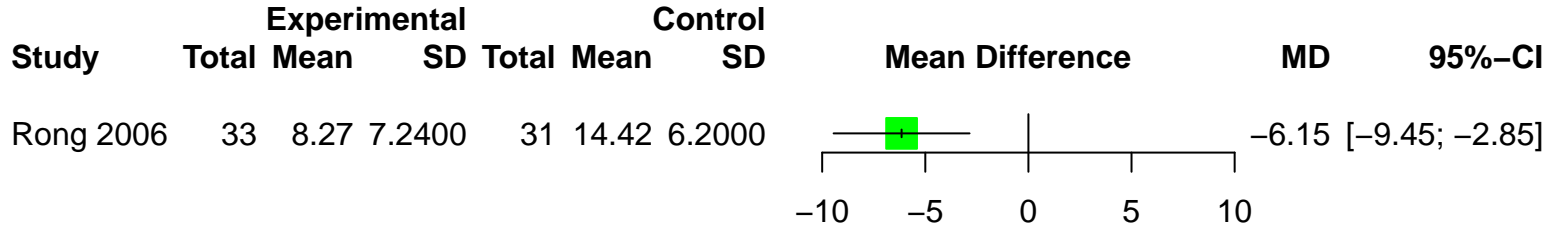

Supplement: Supplementary file 1 [file Data_Sheet_1.ZIP › Supplementary files/Fig S2-3.MHOWS.MAvsEA.pdf]

Trace of d.WM.TEAS

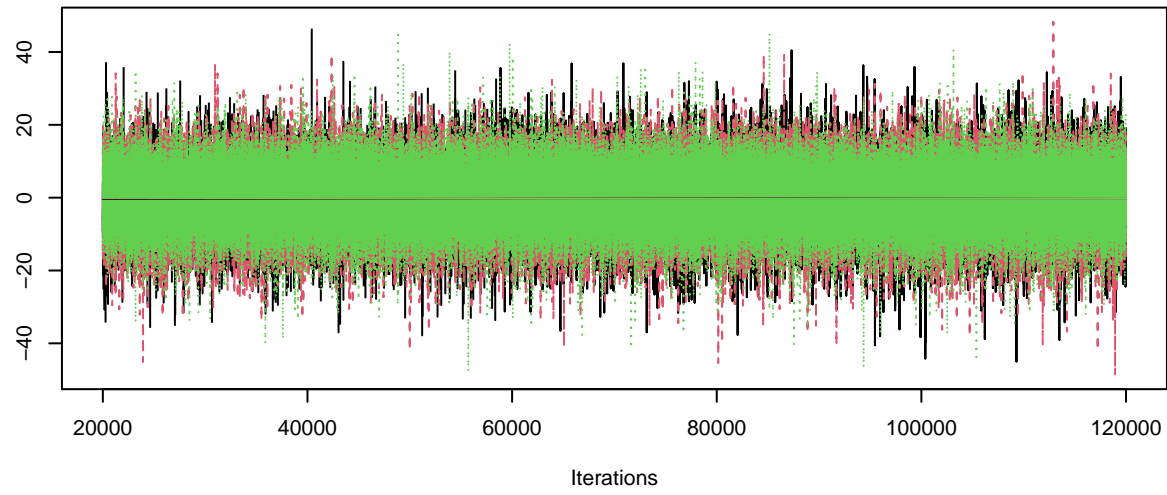

Density of d.WM.TEAS

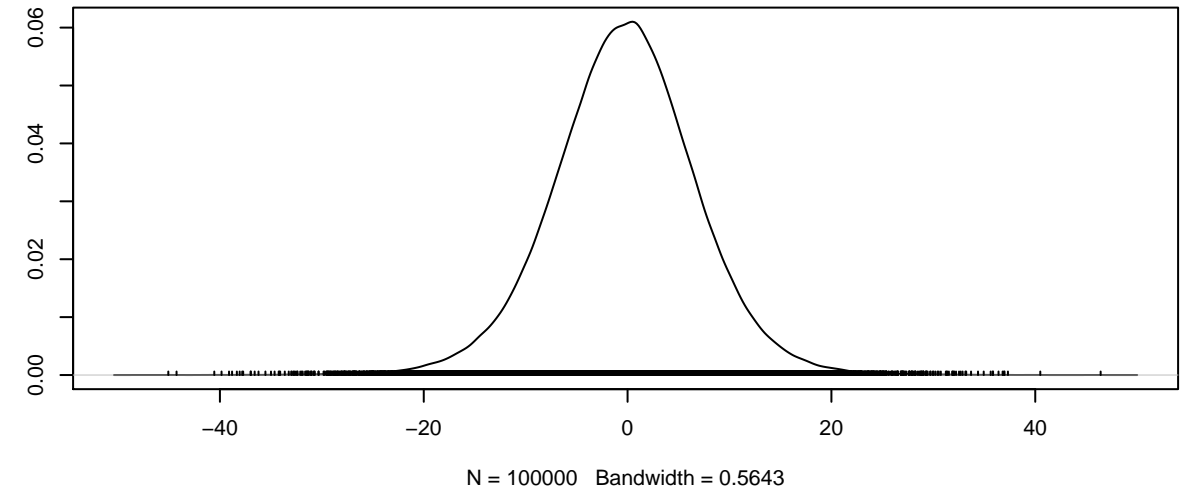

Trace of sd.d

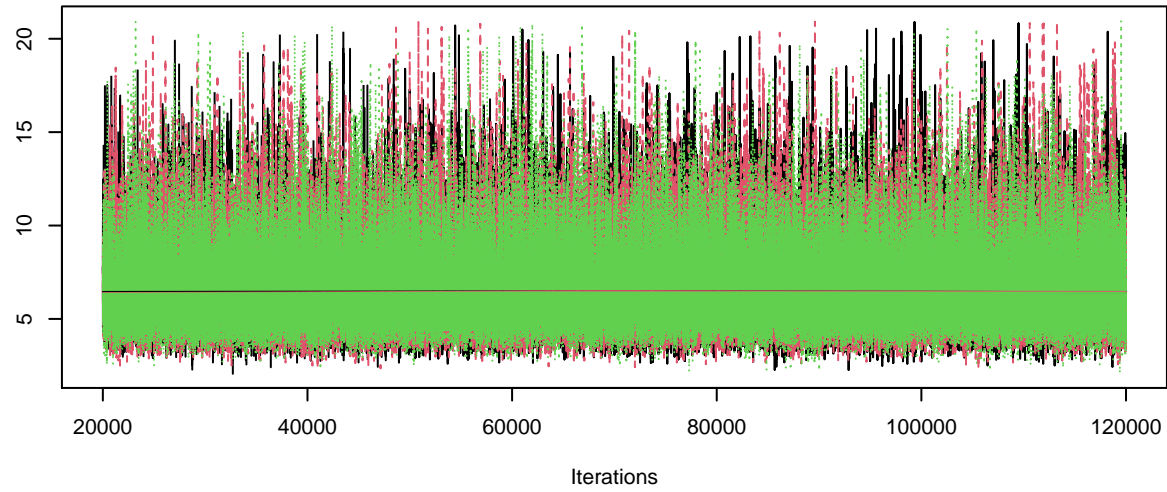

Density of sd.d

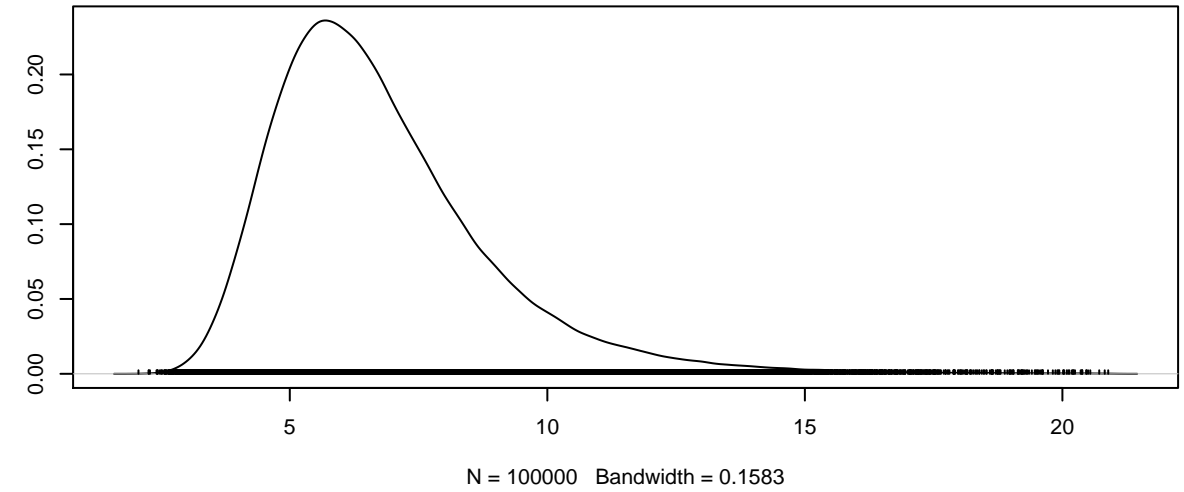

Supplement: Supplementary file 1 [file Data_Sheet_1.ZIP › Supplementary files/Fig S6-2.Trace plot of MHOWS-2.pdf]

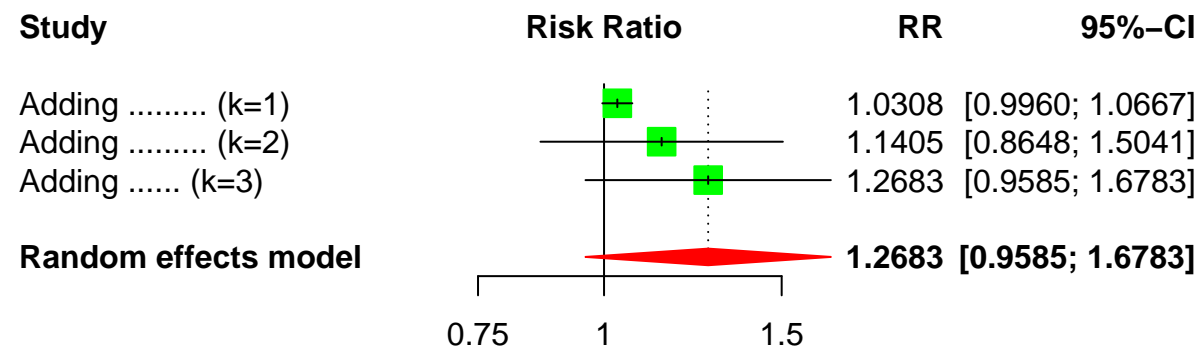

Supplement: Supplementary file 1 [file Data_Sheet_1.ZIP › Supplementary files/Fig S3-3.ER.MAvsWM.pdf]

**Trace of d.WM.AA**

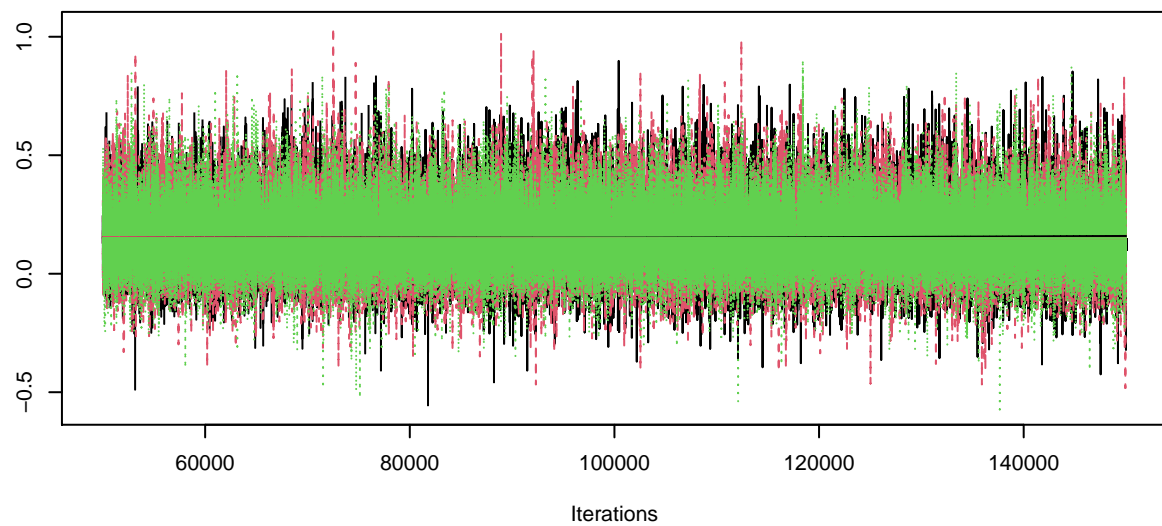

**Density of d.WM.AA**

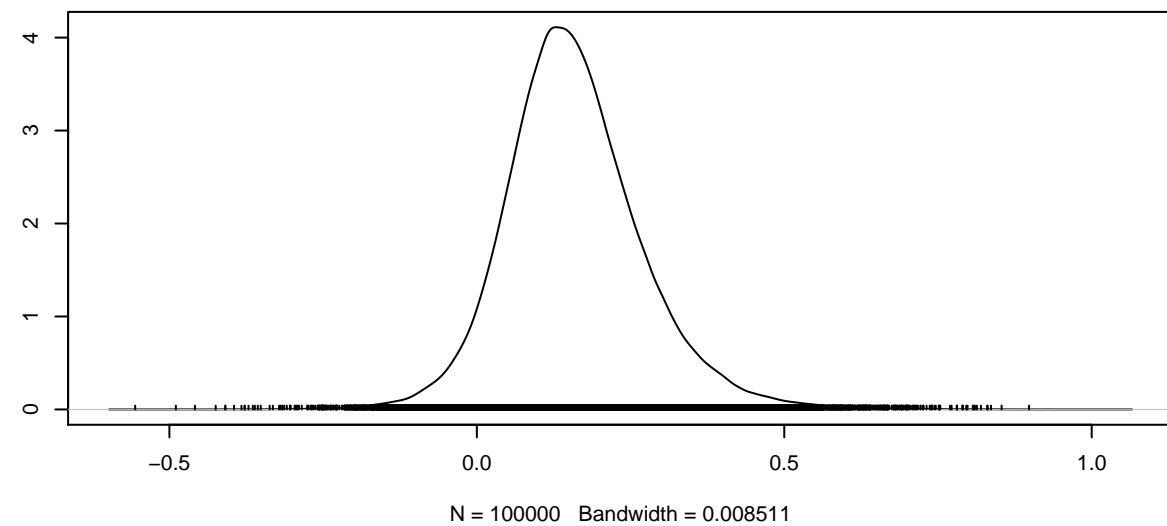

**Trace of d.WM.EA**

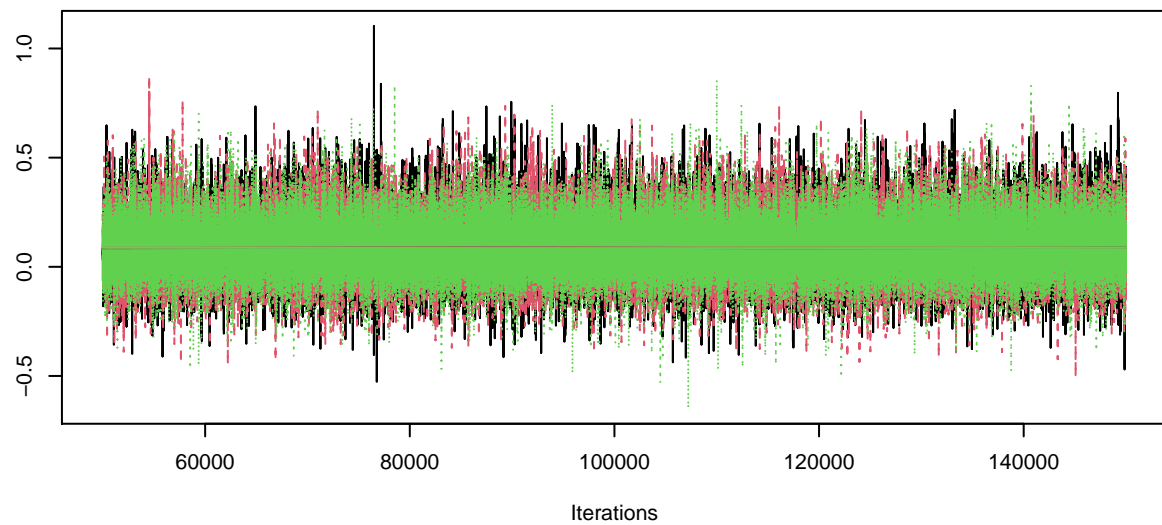

**Density of d.WM.EA**

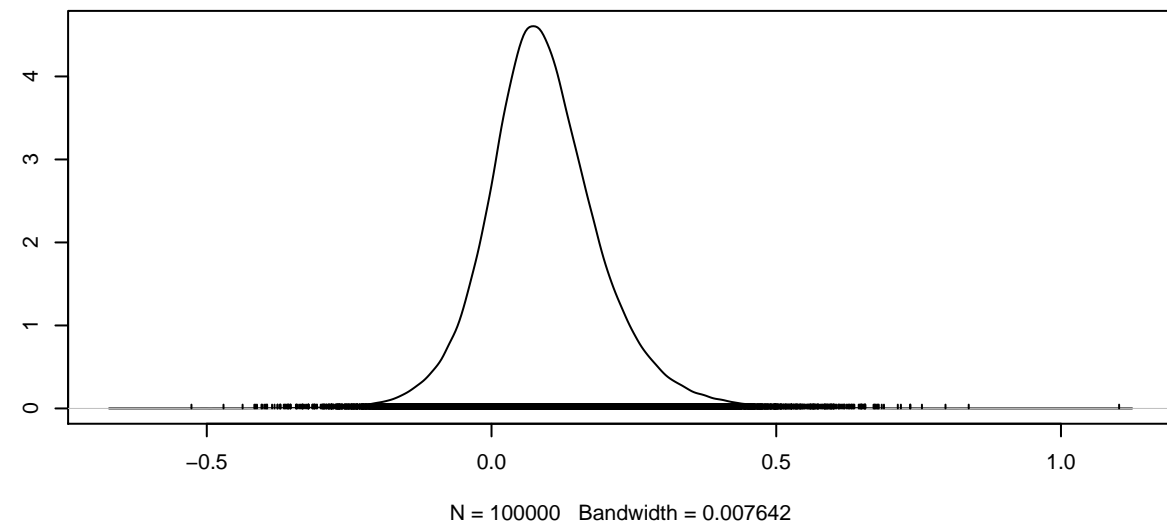

**Trace of d.WM.MA**

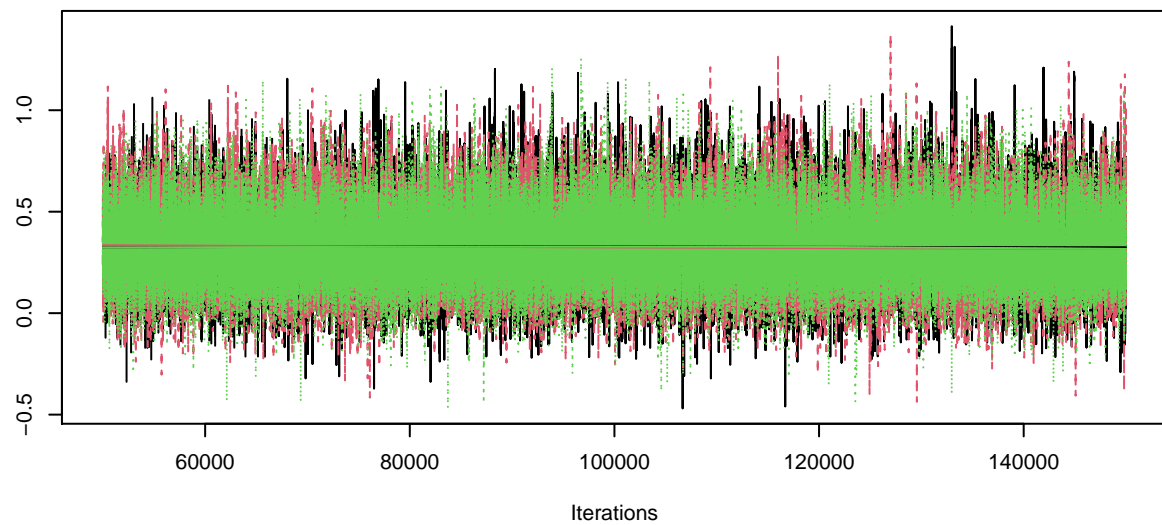

**Density of d.WM.MA**

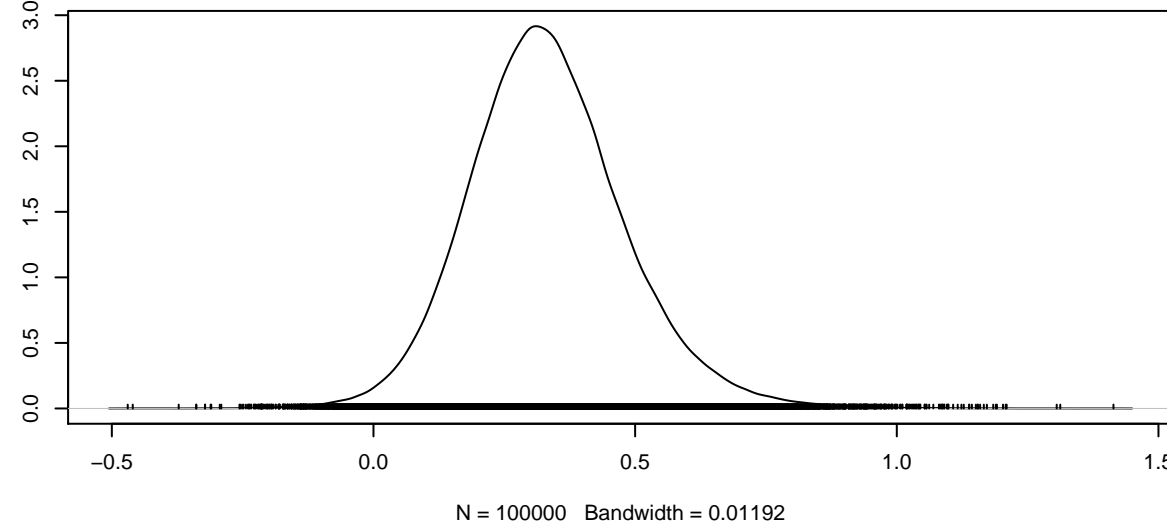

Supplement: Supplementary file 1 [file Data_Sheet_1.ZIP › Supplementary files/Fig S5-1.Trace plot of effective rate-1.pdf]

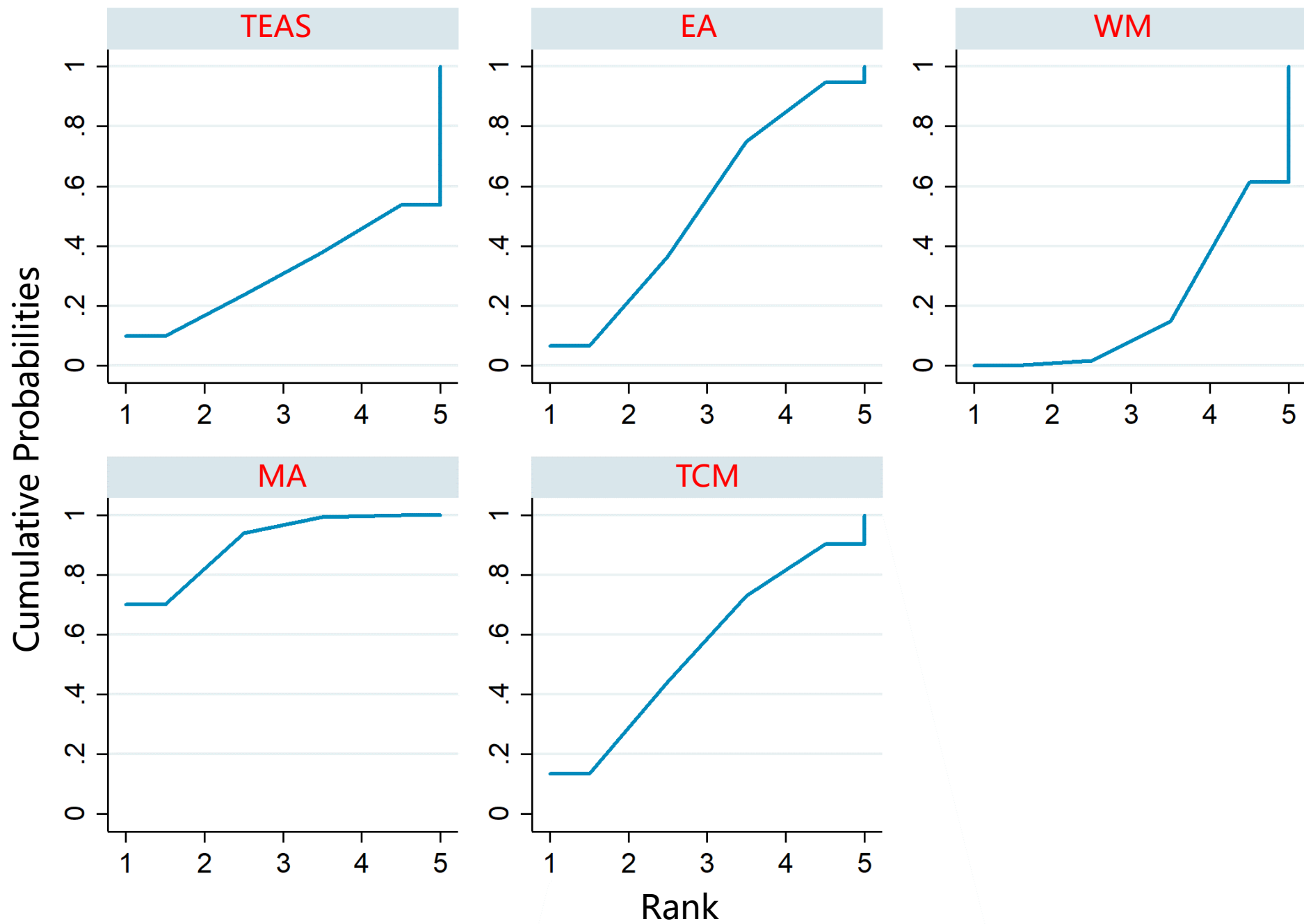

Supplement: Supplementary file 1 [file Data_Sheet_1.ZIP › Supplementary files/Fig S12. SUCRA of MHOWS.pdf]

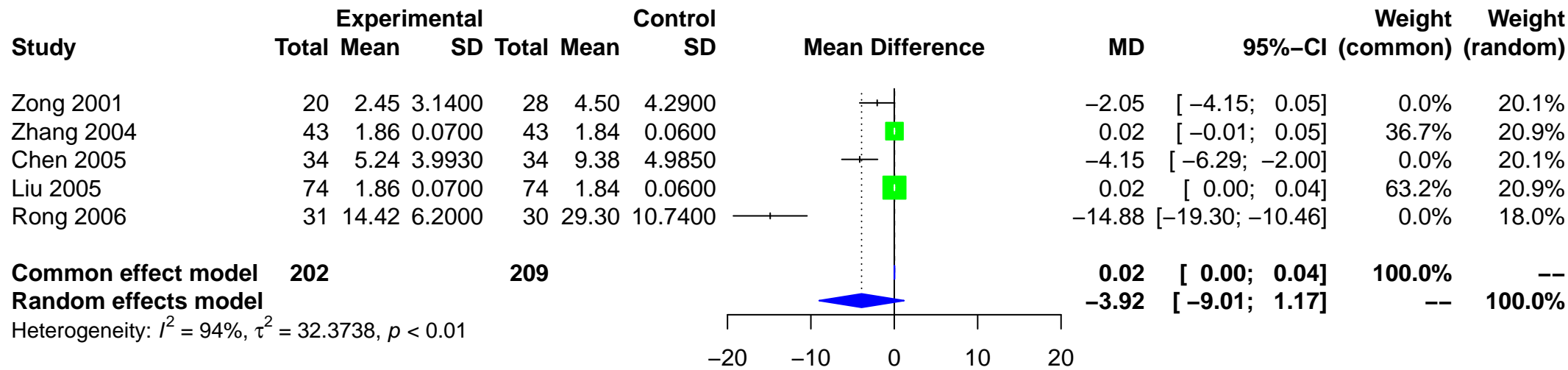

Supplement: Supplementary file 1 [file Data_Sheet_1.ZIP › Supplementary files/Fig S2-2.MHOWS.EAvsWM.pdf]

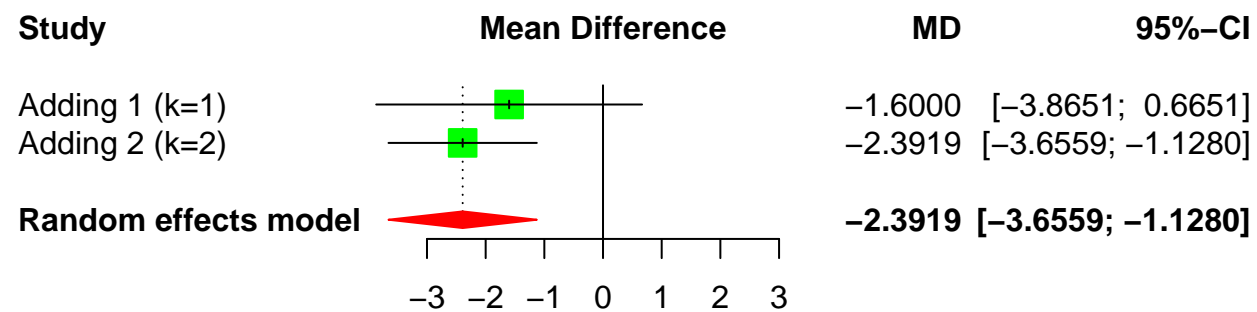

Supplement: Supplementary file 1 [file Data_Sheet_1.ZIP › Supplementary files/Fig S4-3.MHOWS.TCMvsWM.pdf]

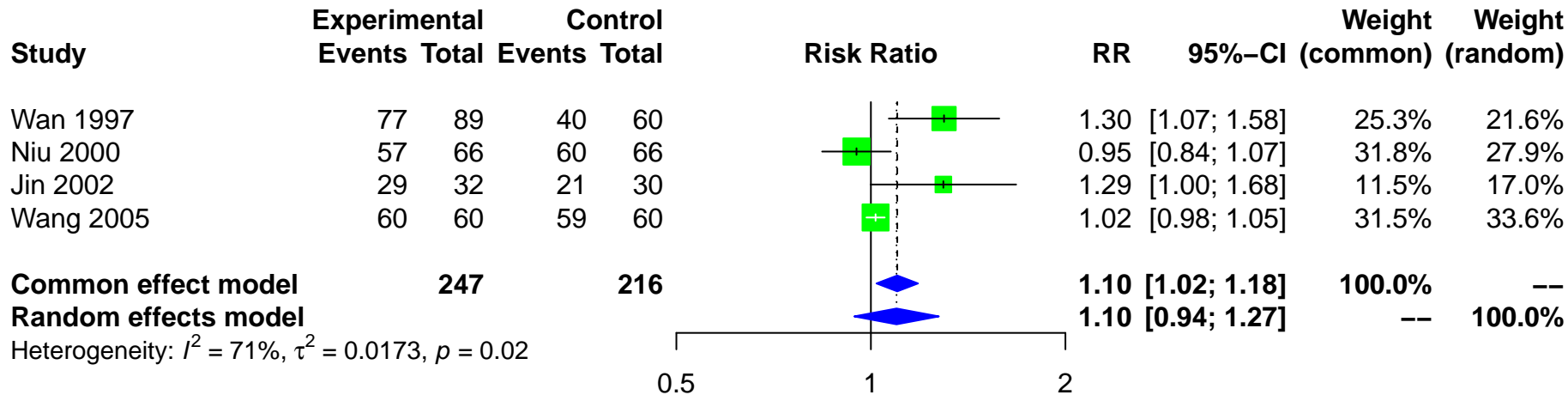

Supplement: Supplementary file 1 [file Data_Sheet_1.ZIP › Supplementary files/Fig S1-1.ER.AAvsWM.pdf]

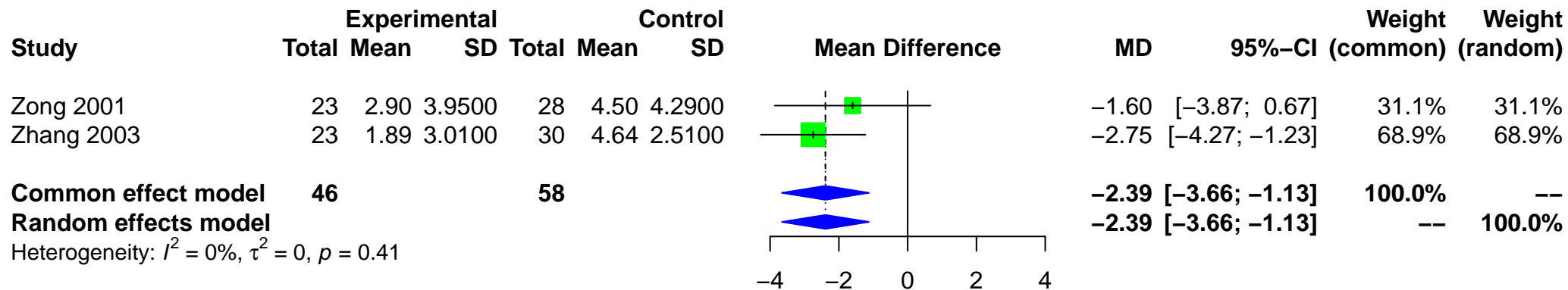

Supplement: Supplementary file 1 [file Data_Sheet_1.ZIP › Supplementary files/Fig S2-7.MHOWS.TCMvsWM.pdf]

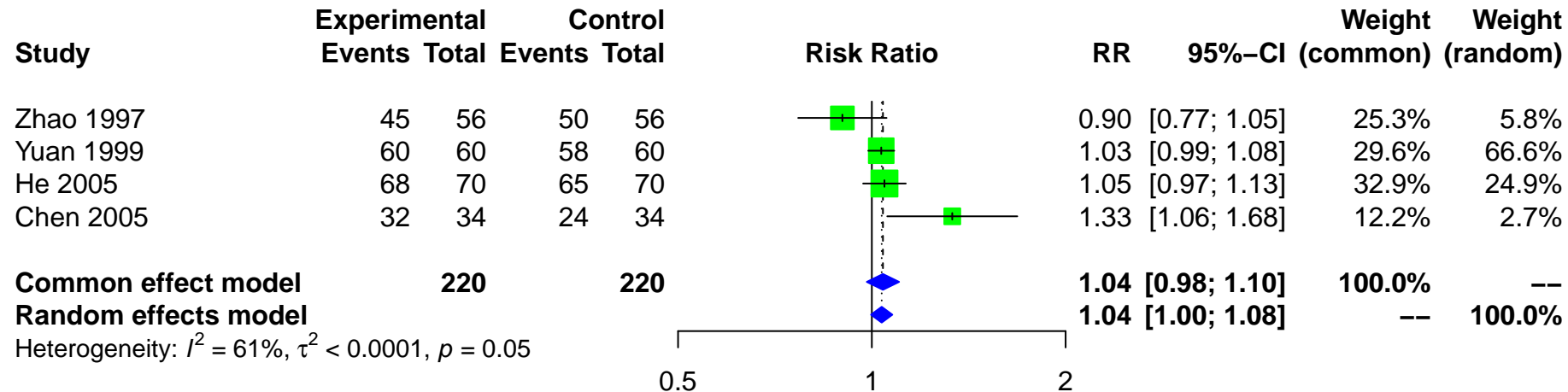

Supplement: Supplementary file 1 [file Data_Sheet_1.ZIP › Supplementary files/Fig S1-3.ER.EAvsWM.pdf]

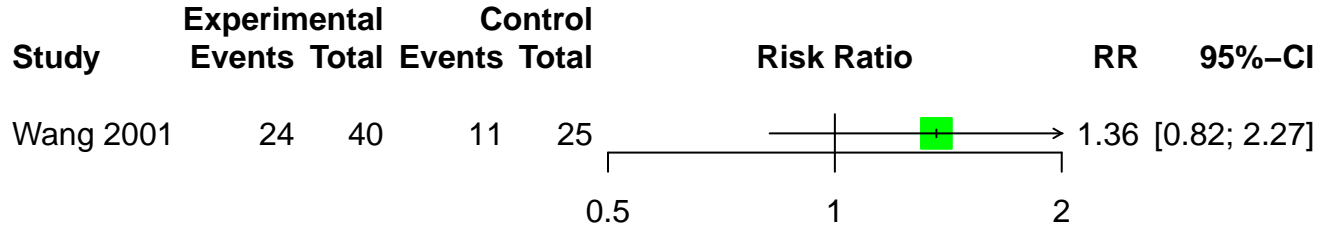

Supplement: Supplementary file 1 [file Data_Sheet_1.ZIP › Supplementary files/Fig S1-7.ER.TEASvsTCM.pdf]

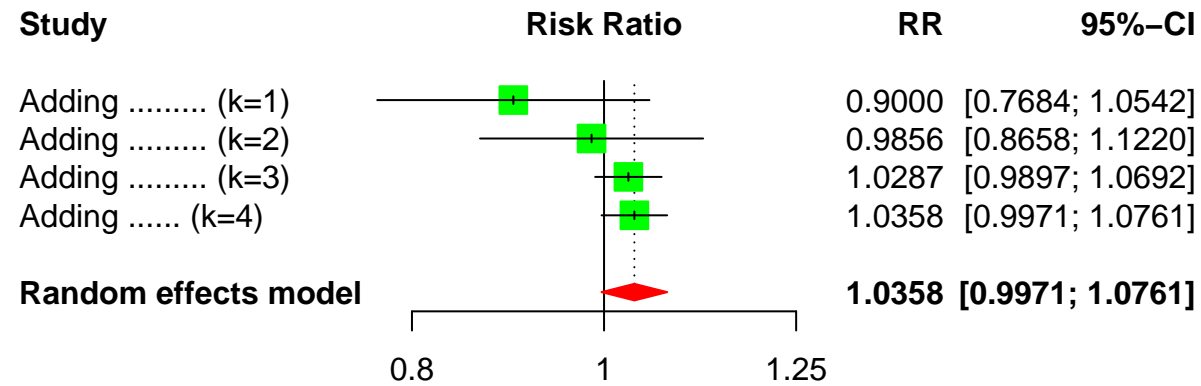

Supplement: Supplementary file 1 [file Data_Sheet_1.ZIP › Supplementary files/Fig S3-2.ER.EAvsWM.pdf]

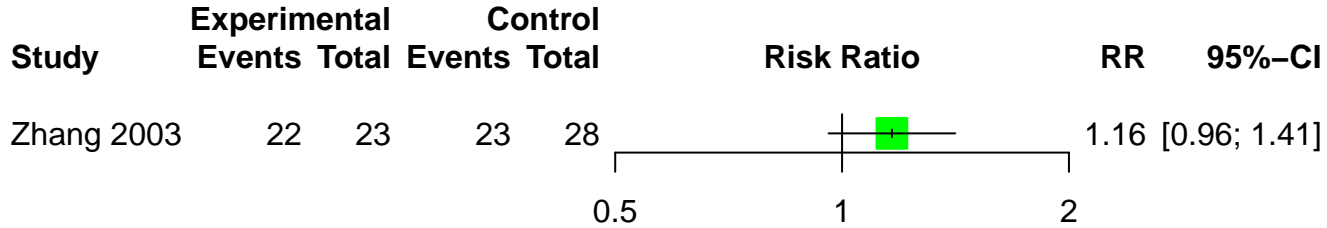

Supplement: Supplementary file 1 [file Data_Sheet_1.ZIP › Supplementary files/Fig S1-4.ER.MAvsTCM.pdf]

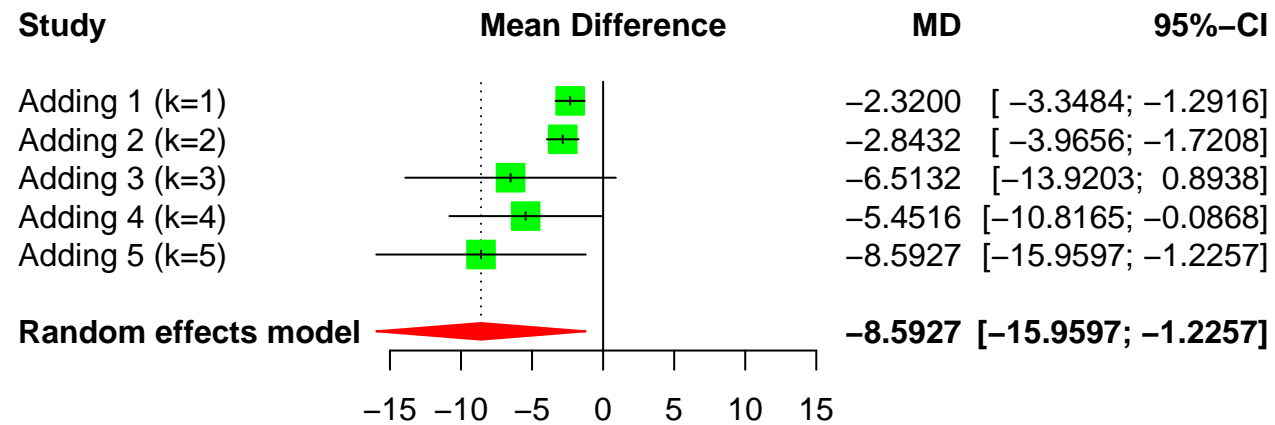

Supplement: Supplementary file 1 [file Data_Sheet_1.ZIP › Supplementary files/Fig S4-2.MHOWS.MAvsWM.pdf]

Cumulative Probabilities

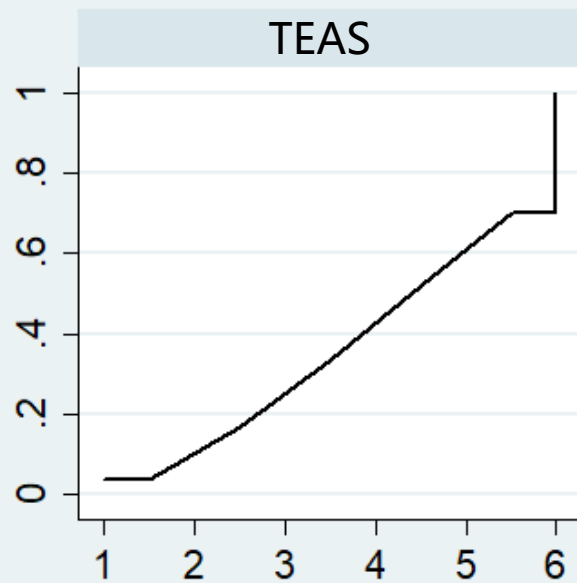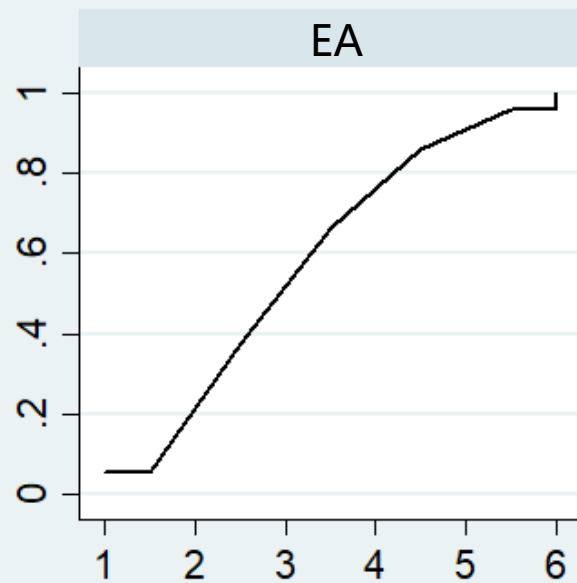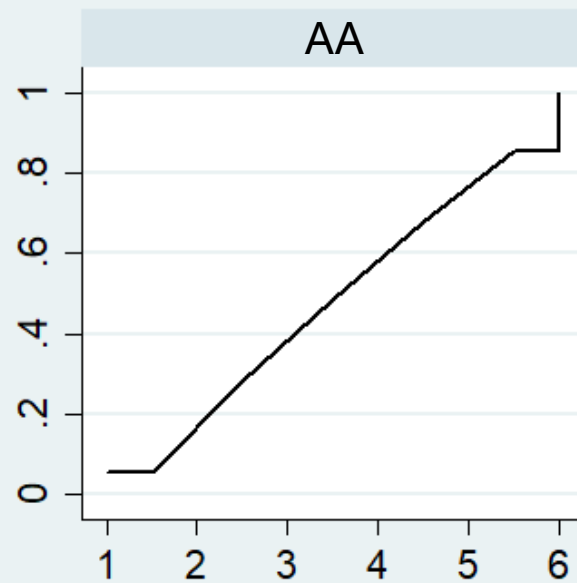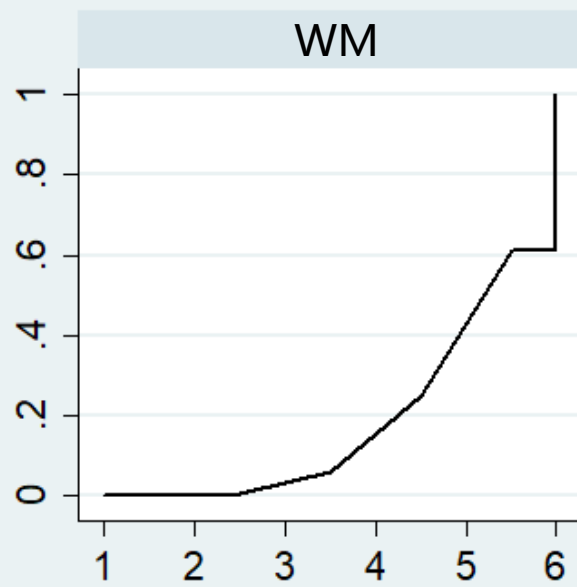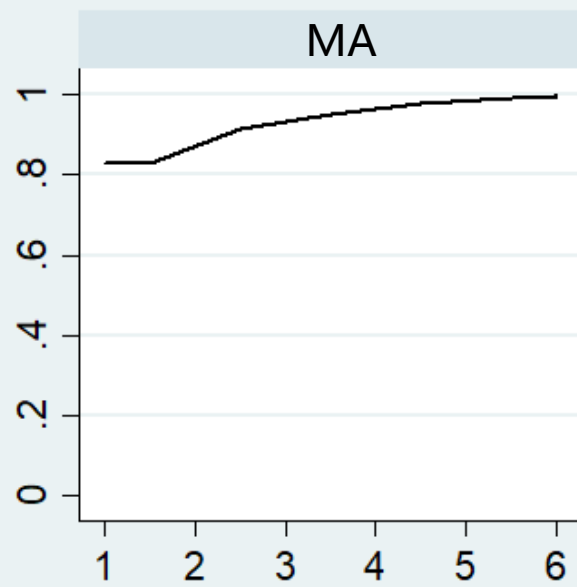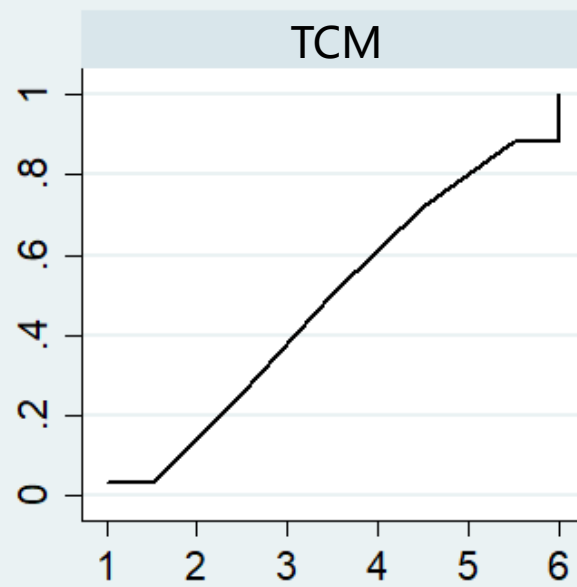

Rank

Supplement: Supplementary file 1 [file Data_Sheet_1.ZIP › Supplementary files/Fig S11. SUCRA of effective rate.pdf]

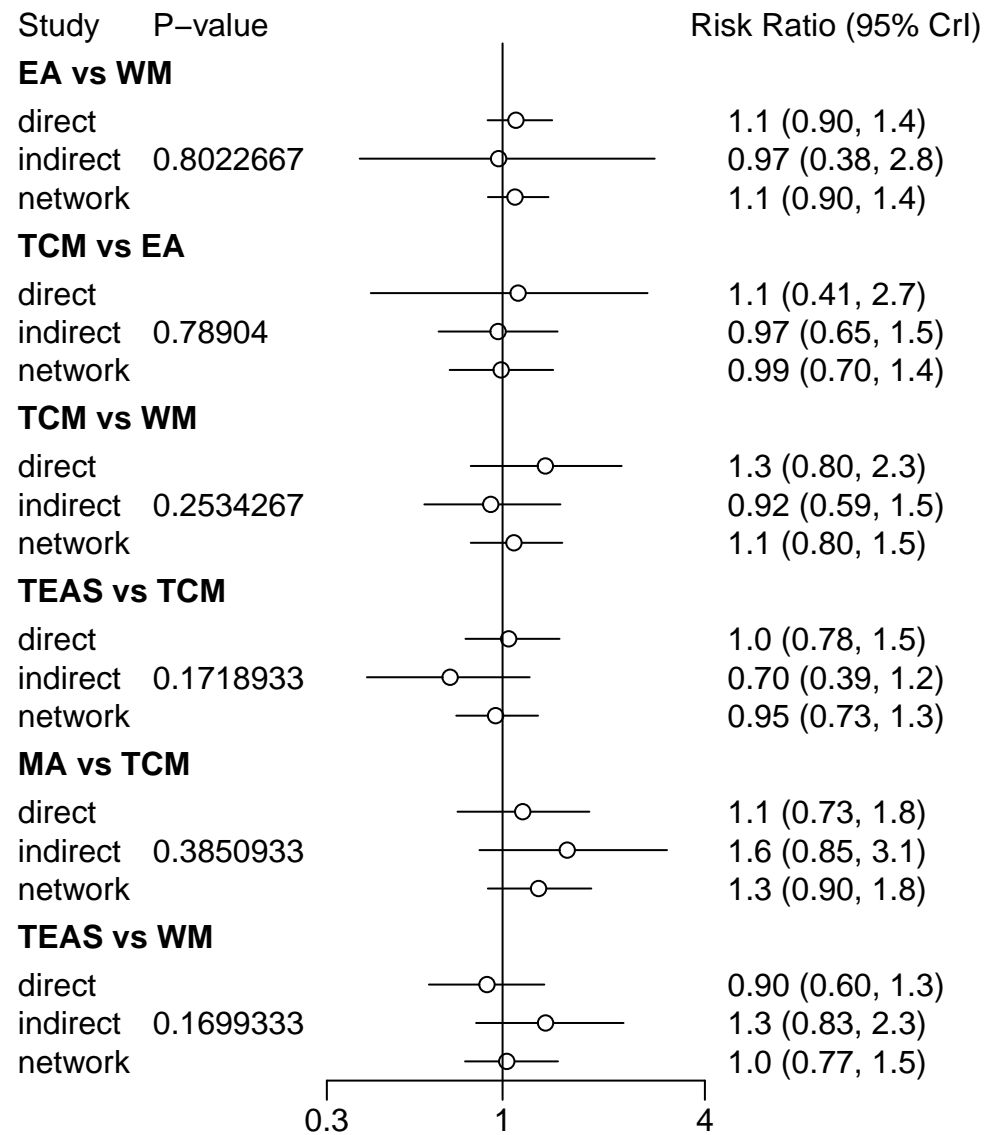

Supplement: Supplementary file 1 [file Data_Sheet_1.ZIP › Supplementary files/Fig S9. Node-splitting plot of effective rate.pdf]

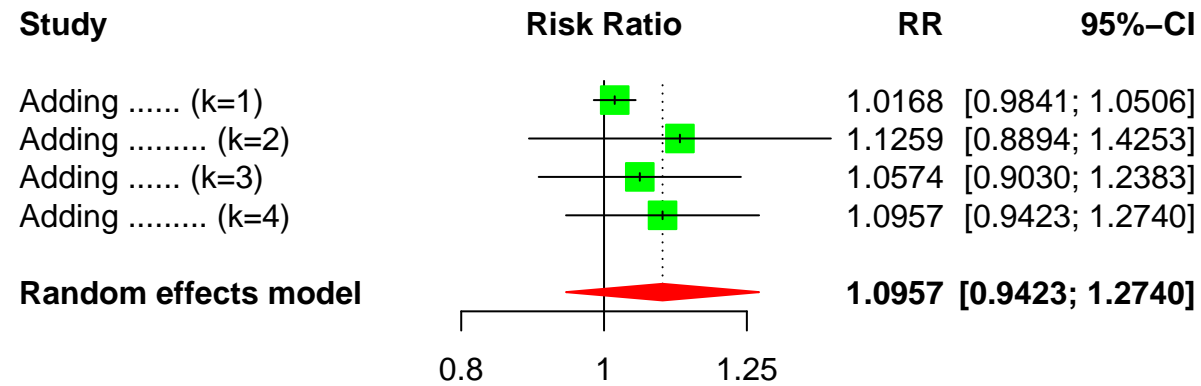

Supplement: Supplementary file 1 [file Data_Sheet_1.ZIP › Supplementary files/Fig S3-1.ER.AAvsWM.pdf]

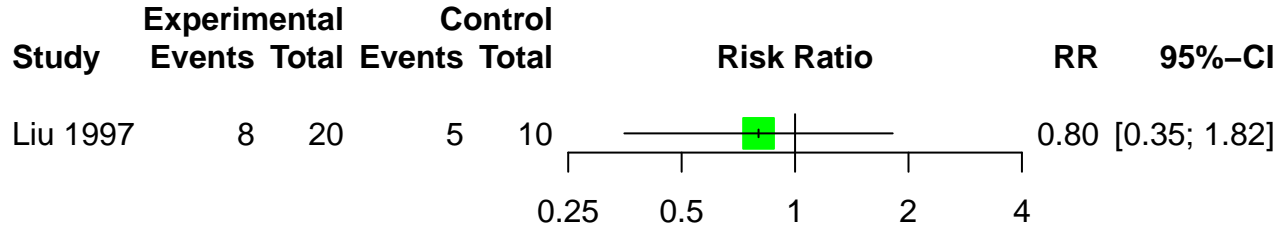

Supplement: Supplementary file 1 [file Data_Sheet_1.ZIP › Supplementary files/Fig S1-2.ER.EAvsTCM.pdf]

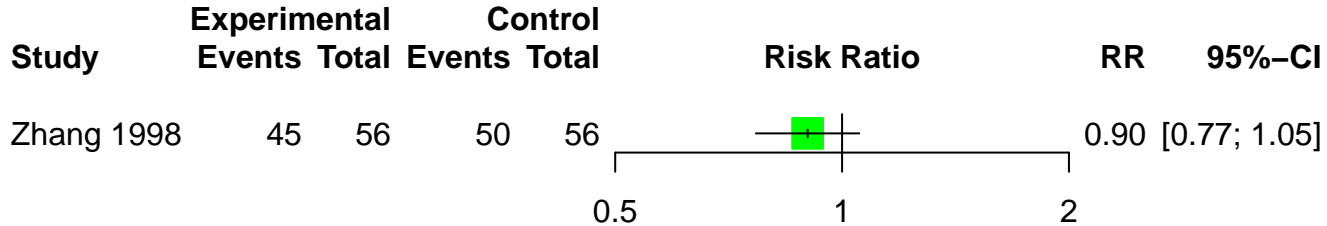

Supplement: Supplementary file 1 [file Data_Sheet_1.ZIP › Supplementary files/Fig S1-8.ER.TEASvsWM.pdf]

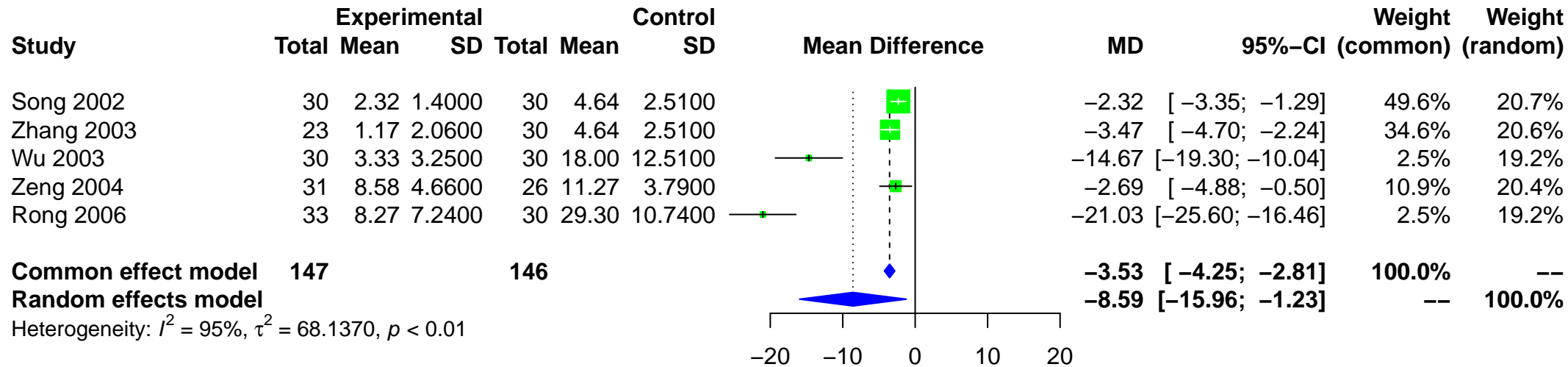

Supplement: Supplementary file 1 [file Data_Sheet_1.ZIP › Supplementary files/Fig S2-6.MHOWS.MAvsWM.pdf]

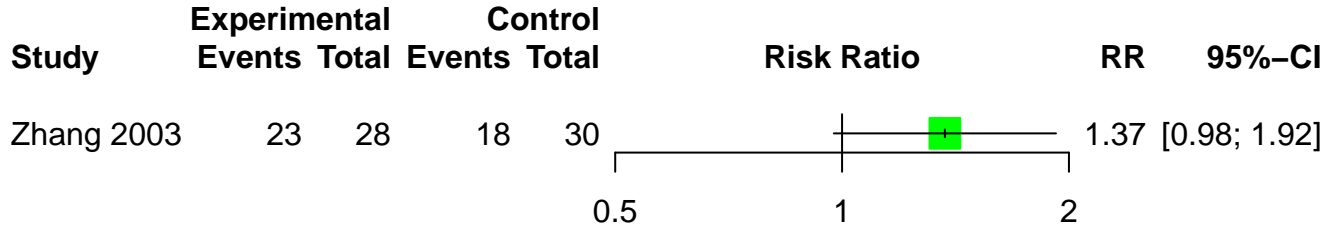

Supplement: Supplementary file 1 [file Data_Sheet_1.ZIP › Supplementary files/Fig S1-6.ER.TCMvsWM.pdf]

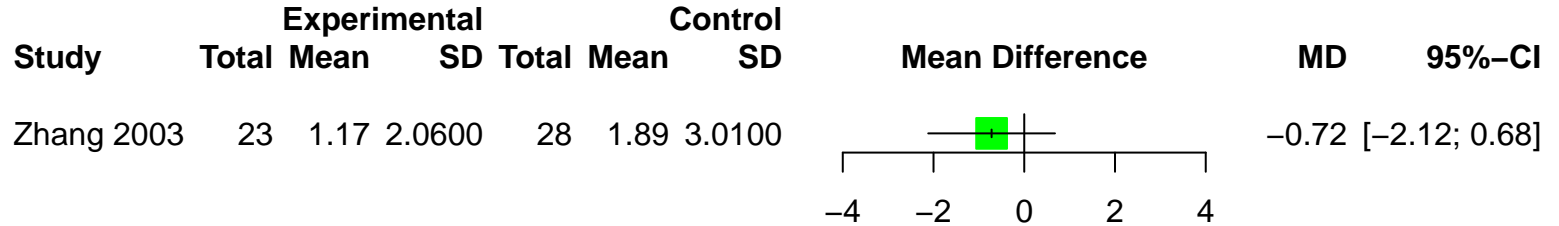

Supplement: Supplementary file 1 [file Data_Sheet_1.ZIP › Supplementary files/Fig S2-4.MHOWS.MAvsTCM.pdf]

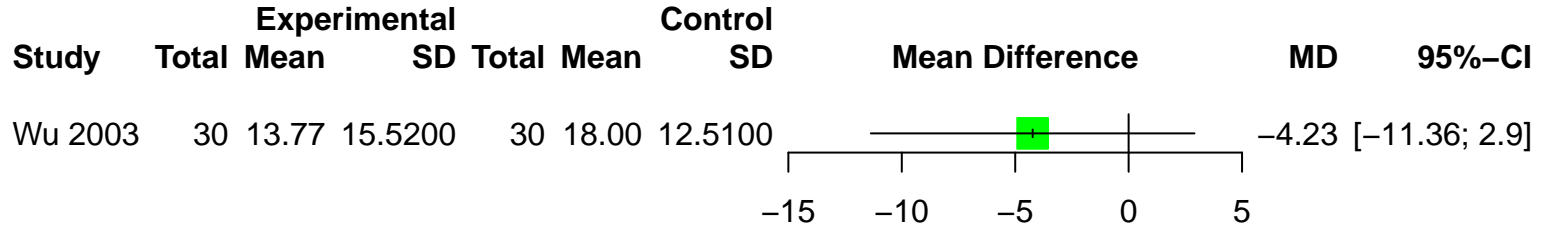

Supplement: Supplementary file 1 [file Data_Sheet_1.ZIP › Supplementary files/Fig S2-8.MHOWS.TEASvsWM.pdf]

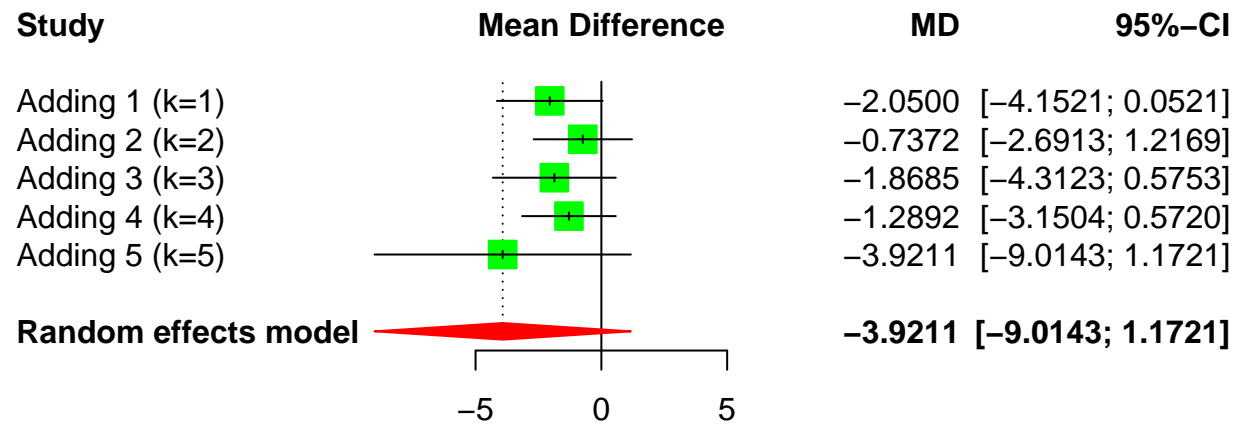

Supplement: Supplementary file 1 [file Data_Sheet_1.ZIP › Supplementary files/Fig S4-1.MHOWS.EAvsWM.pdf]

**d.WM.AA**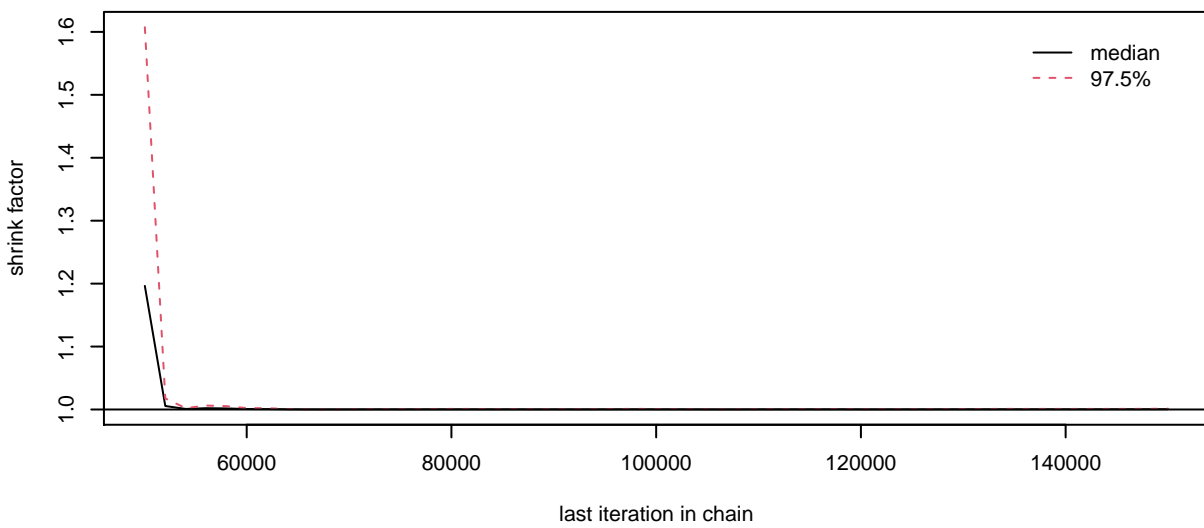**d.WM.EA**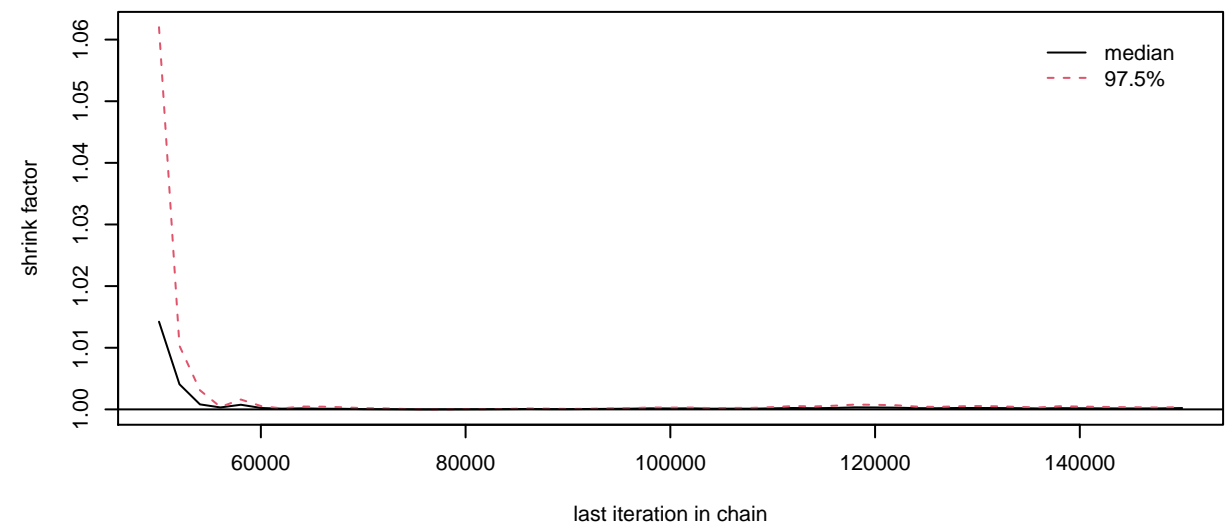**d.WM.MA**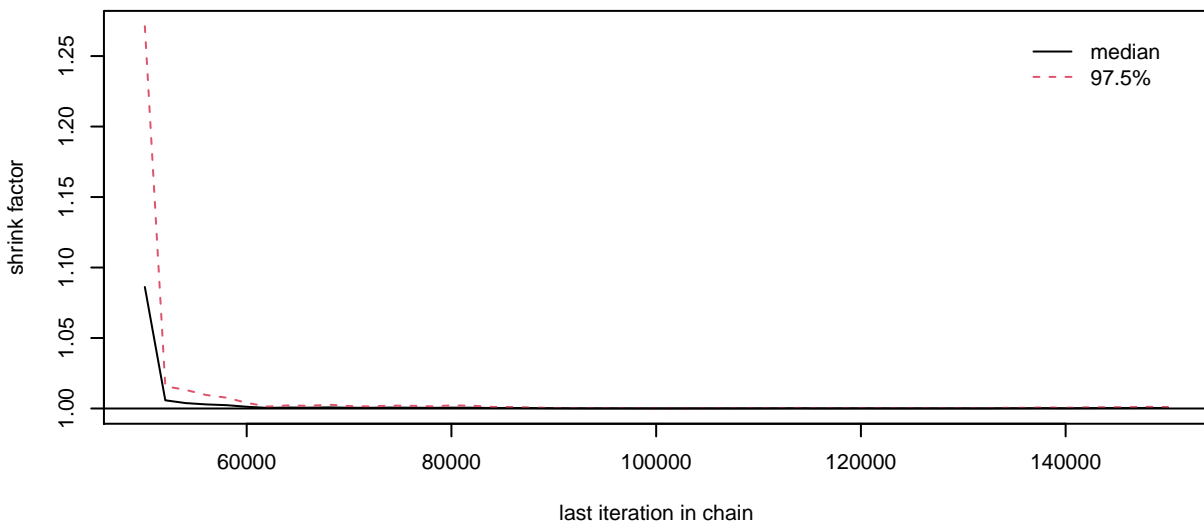**d.WM.TCM**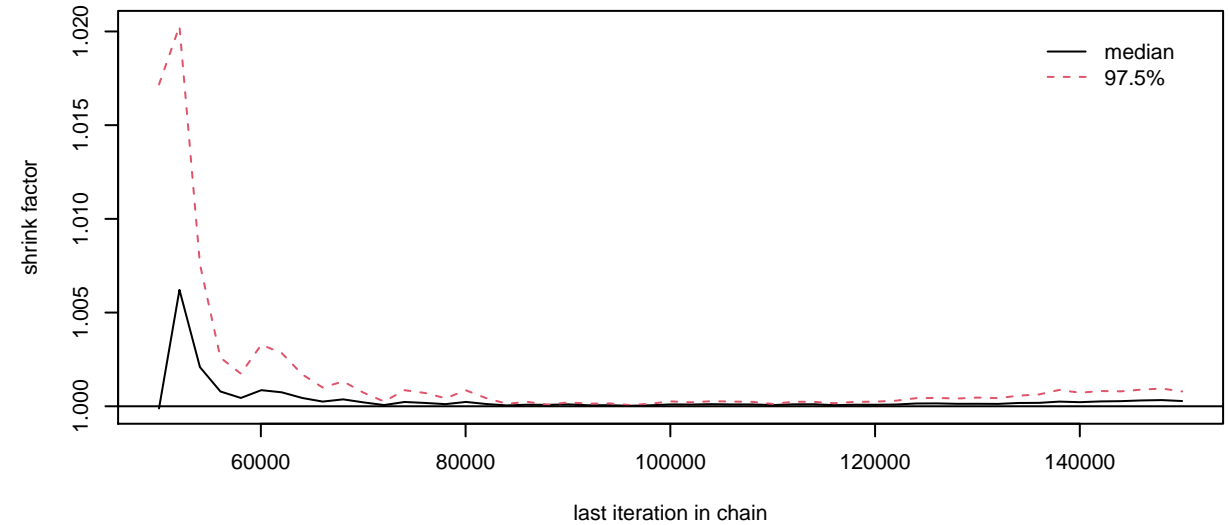**d.WM.TEAS**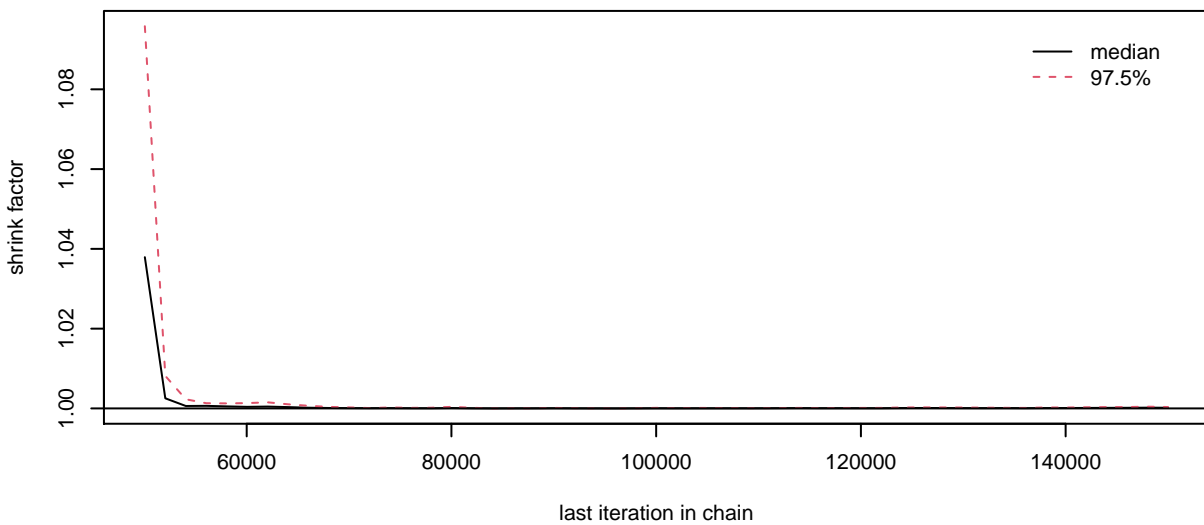**sd.d**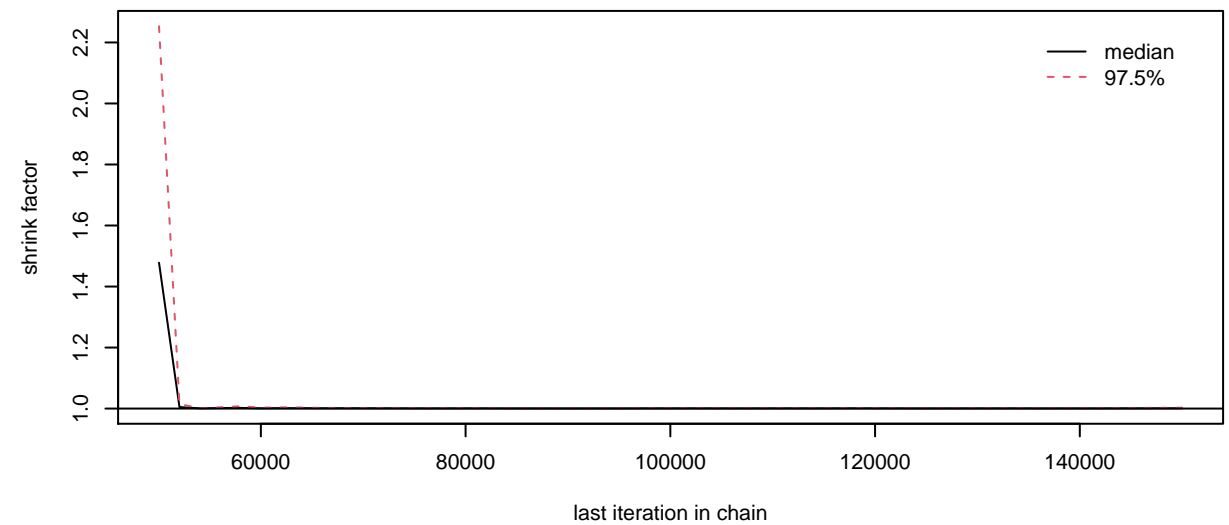

Supplement: Supplementary file 1 [file Data_Sheet_1.ZIP › Supplementary files/Fig S7. Gelman-Rubin-Brooks plot of effective rate.pdf]
